# Subtilase cytotoxin from Shiga-toxigenic *Escherichia coli* impairs the inflammasome and exacerbates enteropathogenic bacterial infection

**DOI:** 10.1016/j.isci.2022.104050

**Published:** 2022-03-10

**Authors:** Hiroyasu Tsutsuki, Tianli Zhang, Kinnosuke Yahiro, Katsuhiko Ono, Yukio Fujiwara, Sunao Iyoda, Fan-Yan Wei, Kazuaki Monde, Kazuko Seto, Makoto Ohnishi, Hiroyuki Oshiumi, Takaaki Akaike, Tomohiro Sawa

**Affiliations:** 1 Department of Microbiology, Graduate School of Medical Sciences, Kumamoto University, 1-1-1 Honjo, Chuo-ku, Kumamoto 860-8556, Japan; 2 Department of Microbiology and Infection Control Sciences, Kyoto Pharmaceutical University, 5 Misasagi-Nakauchi-cho, Yamashina-ku, Kyoto 607-8414, Japan; 3 Department of Cell Pathology, Graduate School of Medical Sciences, Kumamoto University, Chuo-ku, Kumamoto 860-8556, Japan; 4 Department of Bacteriology I, National Institute of Infectious Diseases, 1-23-1 Toyama, Shinjuku-ku, Tokyo 162-8640, Japan; 5 Department of Modomics Biology and Medicine, Institute of Development, Aging and Cancer, Tohoku University, 4-1 Seiryo-machi, Aoba-ku, Sendai 980-8575, Japan; 6 Quality Assurance Unit, Division of Planning, Osaka Institute of Public Health, 1-3-69 Nakamichi, Higashinari-ku, Osaka 537-0025, Japan; 7 Department of Immunology, Graduate School of Medical Sciences, Kumamoto University, Chuo-ku, Kumamoto 860-8556, Japan; 8 Department of Environmental Medicine and Molecular Toxicology, Tohoku University Graduate School of Medicine, 2-1 Seiryo-machi, Aoba-ku, Sendai 980-8575, Japan

**Keywords:** Biochemistry, Protein, Microbiology, Bacteriology

## Abstract

Subtilase cytotoxin (SubAB) is an AB_5_ toxin mainly produced by the locus of enterocyte effacement-negative Shiga-toxigenic *Escherichia coli* (STEC) strain such as O113:H21, yet the contribution of SubAB to STEC infectious disease is unclear. We found that SubAB reduced activation of the STEC O113:H21 infection-induced non-canonical NLRP3 inflammasome and interleukin (IL)-1β and IL-18 production in murine macrophages. Downstream of lipopolysaccharide signaling, SubAB suppressed caspase-11 expression by inhibiting interferon-β/STAT1 signaling, followed by disrupting formation of the NLRP3/caspase-1 assembly. These inhibitions were regulated by PERK/IRE1α-dependent endoplasmic reticulum (ER) stress signaling initiated by cleavage of the host ER chaperone BiP by SubAB. Our murine model of SubAB-producing *Citrobacter rodentium* demonstrated that SubAB promoted *C. rodentium* proliferation and worsened symptoms such as intestinal hyperplasia and diarrhea. These findings highlight the inhibitory effect of SubAB on the NLRP3 inflammasome via ER stress, which may be associated with STEC survival and infectious disease pathogenicity in hosts.

## Highlights

• SubAB from STEC inhibits inflammasome activation and IL-1β/IL-18 production
• SubAB prevents caspase-11 expression via IRE1α/PERK-dependent inhibition of STAT1
• SubAB reduces LPS-induced pro-IL-1β production via IRE1α/PERK-dependent pathway
• SubAB promotes *C. rodentium* survival in mouse colon and facilitates the infection

## Graphical abstract

**Figure:**
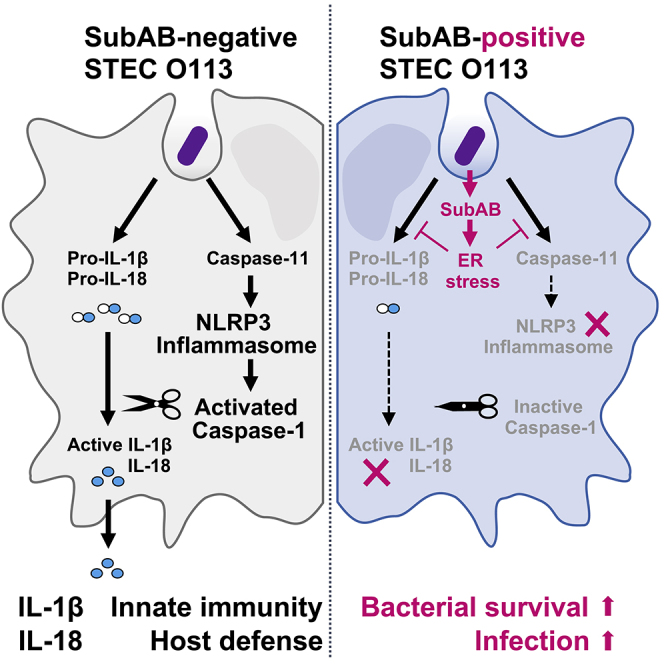

## Introduction

Shiga-toxigenic *Escherichia coli* (STEC) is a major food-borne pathogen that can cause bloody diarrhea and life-threatening hemolytic uremic syndrome; it is associated with outbreaks worldwide. Although the major virulence factors of STEC are Shiga toxins (Stx1 and Stx2), additional virulence factors such as intimin, cytolethal distending toxins, and hemolysin may promote the colonization or pathogenicity of STEC (Krause et al., 2018). Subtilase cytotoxin (SubAB), a member of the AB_5_ toxin family, was identified in the Stx2-producing locus of enterocyte effacement (LEE)-negative STEC O113:H21 strain 98NK2. This STEC serotype was responsible for an outbreak of hemolytic-uremic syndrome in Australia (Paton et al., 2004). SubAB binds to eukaryotic cell surface receptors (Byres et al., 2008; Yahiro et al., 2006, 2011; Yamaji et al., 2019), translocates to the endoplasmic reticulum (ER), and cleaves the ER chaperone BiP/Grp78 (Paton et al., 2006). This BiP cleavage by SubAB triggers the ER stress response mediated by protein kinase R-like ER kinase (PERK), inositol-requiring kinase 1α (IRE1α), and activating transcription factor 6 (ATF6) (Wolfson et al., 2008), which leads to cell death (May et al., 2010; Paton et al., 2006; Wolfson et al., 2008; Yahiro et al., 2010, 2012) and damage in mice including hemorrhagic colitis (Furukawa et al., 2011; Wang et al., 2007, 2011). The *subAB* gene was detected in some LEE-negative STEC strains including clinical human isolates (Fierz et al., 2017; Hoang Minh et al., 2015; Khaitan et al., 2007; Paton et al., 2004), which suggests that SubAB may exacerbate clinical symptoms of STEC infections (Galli et al., 2010; Velandia et al., 2011). However, the role of SubAB in STEC infections remains unclear. Previously, we demonstrated that SubAB inhibited lipopolysaccharide (LPS)-induced nitric oxide (NO) production by suppressing inducible NO synthase (iNOS) expression, which enhanced *E. coli* survival in macrophages (Tsutsuki et al., 2012). Our finding led us hypothesize that SubAB acts as not only a cytotoxin but also an effector protein that disrupts host innate immunity and contributes to a bacterial strategy to elude host defense.

The inflammasome is a multiprotein complex that acts as a platform for activation of caspase-1. Activated caspase-1 proteolytically cleaves the cytosolic sequestering leader sequence of pro-interleukin (IL)-1β and pro-IL-18 to produce and release mature cytokines. IL-1β and IL-18 cause various biological effects associated with cytokine and interferon (IFN) production, which leads to systemic host defense against bacterial infection (Dinarello, 1996; Okamura et al., 1995; van de Veerdonk et al., 2011). The best characterized inflammasome is the Nod-like receptor (NLR) family pyrin domain containing 3 (NLRP3) inflammasome, which comprises NLRP3, the adapter ASC (apoptosis-associated speck-like protein containing a caspase recruitment domain), and pro-caspase-1. In the bacterial infection-induced NLRP3 inflammasome (non-canonical NLRP3 inflammasome), caspase-11 acts as an intracellular LPS sensor and plays a pivotal role in the activation of caspase-1 (Shi et al., 2014). Expression of caspase-11 requires LPS-induced Toll-like receptor 4 signaling through the adaptor TRIF (TIR-domain-containing adaptor-inducing interferon-β) and type I IFN (IFN-α and-β) signaling (Rathinam et al., 2012).

In the present report, we investigated the effects of SubAB on non-canonical inflammasome activation. We found a SubAB-related inhibition mechanism of NLRP3 inflammasome activation and production of IL-1β and IL-18. In addition, we developed a murine infection model for SubAB-producing enteropathogenic bacteria by constructing *Citrobacter rodentium* (*C. rodentium*) bacteria carrying the SubAB-expression plasmid. Our *in vivo* model demonstrated that SubAB enhanced the bacterial burden in the colon. These findings highlight the virulence of SubAB on enteropathogenic bacterial infection. We thus provide direct evidence that enteropathogenic bacteria produce enterotoxin to overcome the host defense system.

## Results

### SubAB inhibits production of IL-1β and IL-18 and activation of inflammasome-related caspases in macrophages

**Figure 1:**
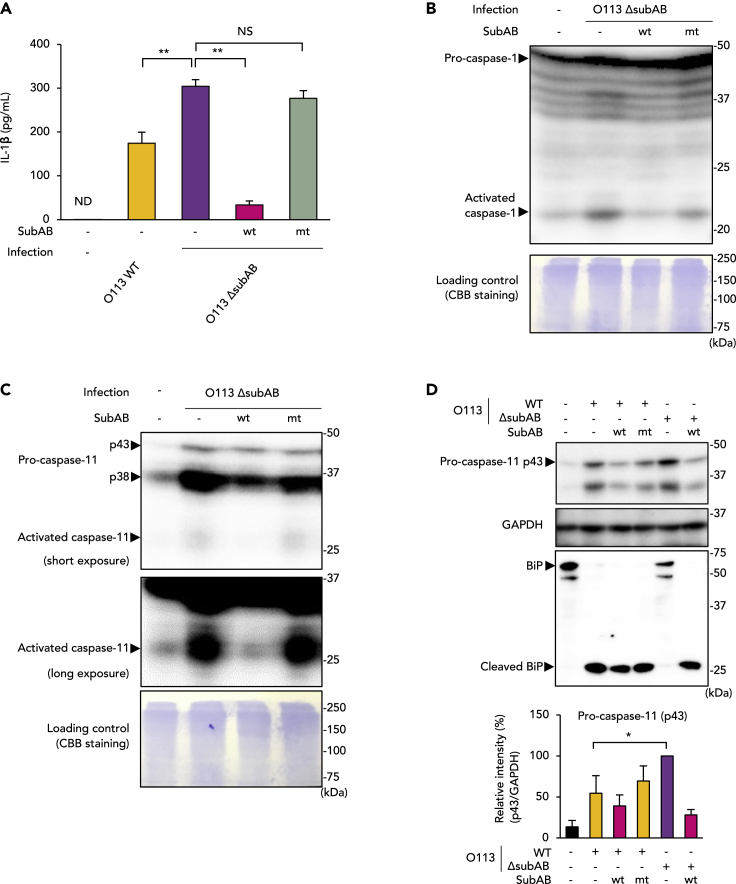
SubAB inhibits production of IL-1β and IL-18 and activation of inflammasome-related caspases in J774.1 cells (A) J774.1 cells were infected with STEC O113 WT or O113 Δ*subAB* (MOI [multiplicity of infection] = 20) in the presence or absence of SubABwt or SubABmt (0.5 μg/mL) for 16 h, and culture supernatants were analyzed for IL-1β by using ELISA. (B and C) Culture supernatants of J774.1 cells infected for 16 h under the indicated condition were concentrated by methanol/chloroform precipitation and were analyzed by Western blotting (WB) using antibody against caspase-1 (B) or caspase-11 (C). CBB, Coomassie brilliant blue. (D) Total cell lysate samples of J774.1 cells infected for 16 h under the indicated conditions were analyzed by using WB with antibodies against caspase-11, GAPDH, or BiP. The bar graph shows a densitometric analysis of the WB of caspase-11 p43. Data are means ± SD (n = 3). ∗p< 0.05; ∗∗p< 0.01; NS, not significant; ND, not detected. See also Figure S1.

To study the effects of SubAB on inflammasome activation, we first investigated whether SubAB affects IL-1β and IL-18 production during infection of macrophages with STEC O113:H21. Infection with *subAB*-positive STEC O113:H21 (wild-type STEC O113:H21 [STEC O113 WT]) resulted in release of IL-1β and IL-18 from murine macrophage cell line J774.1 cells (Figures 1A and S1A). Deletion of the *subAB* gene enhanced production of IL-1β and IL-18 even more, as demonstrated in the experiment on *subAB*-deficient STEC O113: H21 (STEC O113 Δ*subAB*) infection. In the presence of recombinant wild-type SubAB (SubABwt), STEC O113 Δ*subAB*-induced IL-1β and IL-18 production was inhibited, whereas the presence of the catalytically inactivated mutant SubAB (SubABmt) resulted in no inhibition.

Caspase-1 is directly involved in processing pro-IL-1β and pro-IL-18. Caspase-11 regulates the activation of caspase-1 in the non-canonical inflammasome during Gram-negative bacterial infection (Rathinam et al., 2012). We thus next studied the effect of SubAB on the activation of these caspases. STEC O113 Δ*subAB* induced caspase-1 and -11 activation in J774.1 cells, which led to the release of activated caspase-1 (Figure 1B) and caspase-11 (Figure 1C) into culture supernatants. In the presence of SubABwt, activation of both caspase-1 and -11 was inhibited. Because caspase-11 is expressed as pro-caspase-11 before activation (Kayagaki et al., 2011), we investigated intracellular pro-caspase-11 expression. Infection of J774.1 cells with STEC O113 WT induced expression of pro-caspase-11 p43, whereas deletion of the *subAB* gene enhanced pro-caspase-11 p43 expression, as demonstrated in the experiment on STEC O113 Δ*subAB* infection (Figure 1D, upper panel and bar graph). Treatment of J774.1 cells with SubABwt during STEC O113 Δ*subAB* infection suppressed pro-caspase-11 p43 expression. Inasmuch as SubAB cleaves BiP and induces ER stress, we next confirmed BiP cleavage in the infection experiment. Infection of J774.1 cells with STEC O113 WT resulted in marked production of cleaved BiP, whereas STEC O113 Δ*subAB* failed to induce BiP cleavage (Figure 1D, upper panel). Treatment of J774.1 cells with SubABwt during STEC O113 Δ*subAB* infection resulted in BiP cleavage. J774.1 cells infected with STEC O113 WT showed decreased production of IL-1β and IL-18 and activation of caspases, but the number of bacteria present in the cells was higher than that in cells infected with STEC O113 Δ*subAB* (Figure S1B). To test the effect of another ER stress inducer on cytokine production, we used the chemical ER stress inducer tunicamycin (Tm; 1 or 10 μg/mL). During STEC O113 Δ*subAB* infection, Tm also inhibited IL-1β production (Figure S1C). These data suggest that STEC O113-derived SubAB inhibits non-canonical inflammasome activation with concomitant reduction of IL-1β and IL-18 generation through BiP cleavage-mediated ER stress. Caspase-11 cleaves gasdermin D (GSDMD), and the cleaved N-terminal fragment of GSDMD oligomerizes to form pores in the plasma membrane, which leads to pyroptotic cell death (Kayagaki et al., 2015; Shi et al., 2015). Infection with STEC O113 WT increased the cleaved form of GSDMD in J774.1 cells, whereas the cleavage was further promoted during infection with STEC O113 Δ*subAB* (Figure S1D). SubABwt inhibited STEC O113 Δ*subAB*-induced GSDMD cleavage. In agreement with these data, SubAB reduced cell death, as evaluated by the lactate dehydrogenase (LDH) release assay (Figure S1E). These findings suggest that SubAB inhibits pyroptotic cell death in macrophages by caspase-11 inhibition through GSDMD cleavage suppression.

### SubAB prevents LPS-induced pro-IL-1β expression by the PERK- and IRE1α-dependent pathways

**Figure 2:**
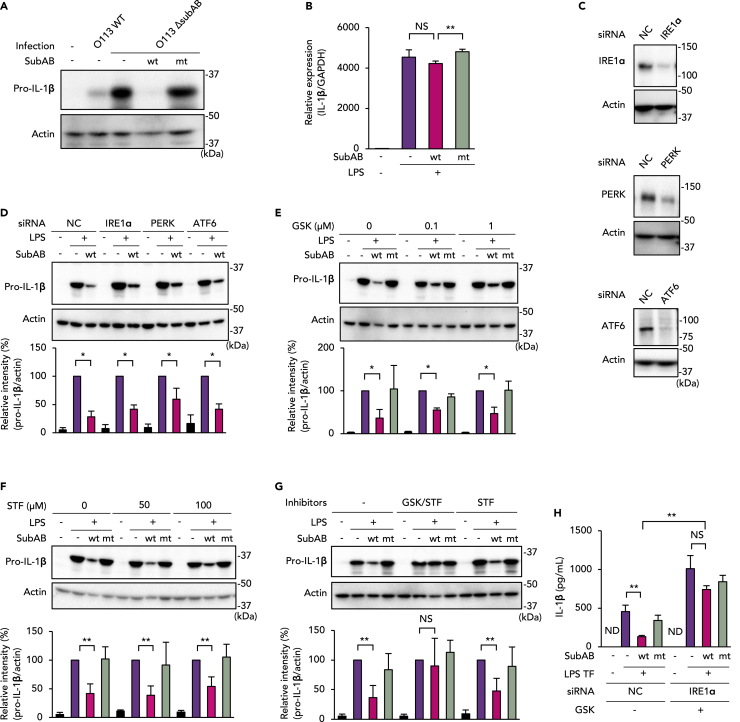
SubAB inhibits pro-IL-1β expression via PERK- and IRE1α-dependent pathways in J774.1 cells (A) Cells were infected with STEC O113 WT or O113 Δ*subAB* (MOI = 20) for 16 h in the presence or absence of SubABwt or SubABmt. Total cell lysate samples were analyzed by WB with anti-IL-1β and anti-actin antibodies. (B) Cells were treated with LPS (100 ng/mL) for 4 h with or without SubABwt or SubABmt. Total RNA extracted from the cells was subjected to qRT-PCR with primers for IL-1β and GAPDH. (C) Different siRNAs—negative control (NC), IRE1α, PERK, or ATF6—were transfected into J774.1 cells. Cell lysates were subjected to WB with anti-IRE1α,-PERK,-ATF6, or-actin antibodies. (D) siRNA-transfected cells were treated with LPS for 4 h with or without SubABwt. (E) Cells were pre-treated with 0.1 or 1 μM GSK2656157 (GSK) for 1 h and were then treated with LPS for 4 h with or without SubABwt or SubABmt. (F) Cells were pre-treated with 50 or 100 μM STF-083010 (STF) for 1 h and were then treated with LPS for 4 h with or without SubABwt or SubABmt. (G) Cells were pre-treated with 1 μM GSK or 100 μM STF and were then treated with LPS for 4 h with or without SubABwt or SubABmt. Total cell lysate samples were analyzed by using WB with anti-IL-1β or-actin antibodies. Band intensity in each case was analyzed by densitometry, and results appear under the WB images (D–G). (H) Negative control (NC) or IRE1α knockdown cells were treated with 1 μM GSK for 1 h, followed by LPS transfection (LPS TF) with or without SubABwt or SubABmt as indicated in method details and Figure S3A. Culture supernatants were subjected to ELISA for IL-1β. Data are means ± SD (n = 3). ∗p< 0.05; ∗∗p< 0.01; NS, not significant. See also Figures S2 andS3.

We studied the inhibition of inflammasome activation by SubAB in greater detail by using an LPS-induced pro-IL-1β expression model. As Figures 2A and S2Ashow, STEC O113 WT infection led to expression of pro-IL-1β. STEC O113 Δ*subAB* infection, however, resulted in much stronger expression of intracellular pro-IL-1β. The expression of pro-IL-1β induced by STEC O113 Δ*subAB* infection was almost completely inhibited by co-treatment with SubABwt (Figure 2A) and the ER stress inducers Tm or thapsigargin (TG) (Figure S2A). As seen in Figure S2B, LPS treatment induced pro-IL-1β expression, which was inhibited by pre-treatment with SubABwt, Tm, or TG. We next analyzed LPS-induced IL-1β mRNA expression in the presence or absence of SubAB. LPS treatment induced transcription of IL-1β mRNA (Figure 2B). Co-treatment with SubABwt slightly reduced LPS-induced IL-1β mRNA; the difference was not statistically significant, however. SubABmt did not alter such LPS-induced IL-1β mRNA results. These data suggested that SubAB may inhibit pro-IL-1β expression during the translation process. To clarify the involvement of ER stress on reduced pro-IL-1β expression by SubAB, we established ER stress sensor knockdown cells (PERK, IRE1α, or ATF6) by small interfering RNA (siRNA) (Figure 2C). Figure 2D illustrates suppression of LPS-induced pro-IL-1β expression in the presence of SubABwt. PERK knockdown somewhat restored pro-IL-1β expression. Pre-treatment with a PERK inhibitor (GSK2656157; GSK) or an IRE1α inhibitor (STF-083010; STF) also slightly attenuated SubABwt-mediated inhibition of LPS-induced pro-IL-1β expression (Figures 2E and 2F). Combination treatment with GSK and STF clearly abolished the inhibitory effect of SubABwt on LPS-induced pro-IL-1β expression (Figures 2G and S2C). Also, double knockdown of IRE1α and PERK nullified the inhibitory effect of SubABwt on pro-IL-1β expression (Figure S2D). Intracellular transfection of LPS (LPS TF) into LPS-primed J774.1 cells induced IL-1β release into the culture supernatant (Figures 2H, S2E, and S3A). Treatment with SubABwt at 3 h after LPS priming inhibited LPS TF-induced IL-1β release, but SubABmt treatment produced no inhibition. In addition, as expected, in IRE1α knockdown cells GSK abolished the inhibitory effect of SubABwt on LPS TF-induced IL-1β release (Figure 2H). Double knockdown of IRE1α and PERK counteracted the inhibitory effect of SubABwt on LPS TF-induced IL-1β release (Figure S2E). These results indicate that SubAB inhibits LPS-induced pro-IL-1β translation that depends on both the PERK and the IRE1α signaling pathways.

### SubAB prevents non-canonical inflammasome-dependent IL-1β release by reducing pro-caspase-11 expression

**Figure 3:**
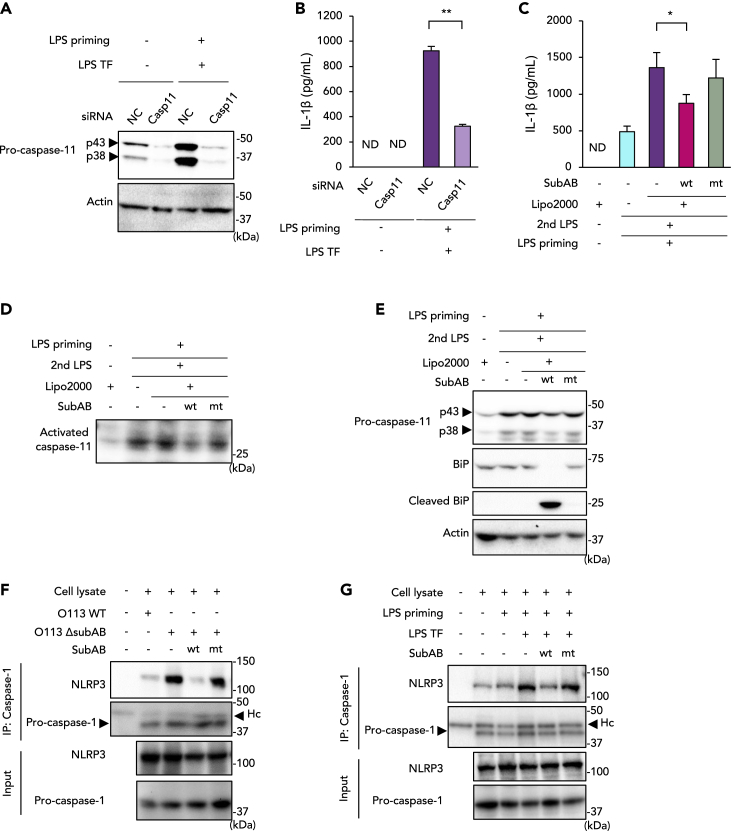
SubAB inhibits formation of the NLRP3 inflammasome complex by suppressing caspase-11 expression in J774.1 cells (A) Gene knockdown of caspase-11 (Casp11) by siRNA. Cells were transfected for 72 h with NC or caspase-11 siRNA. After LPS priming, cells were transfected with LPS as indicated in method details and Figure S3A. (B) IL-1β production by LPS transfection (LPS TF) in culture supernatants of caspase-11 knockdown cells. Culture supernatants were subjected to ELISA to detect IL-1β. (C–E) Suppression by SubAB of LPS TF-induced IL-1β production (C), caspase-11 activation (D), and pro-caspase-11 expression (E). (F) Cells were infected with STEC O113 WT or O113 Δ*subAB* (MOI = 20) for 16 h in the presence or absence of SubABwt or SubABmt. Total cell lysate samples were analyzed by means of immunoprecipitation (IP) with anti-caspase-1 antibody. NLRP3 binding was analyzed via WB with anti-NLRP3 antibody. (G) Cells were stimulated with LPS TF in the presence or absence of SubABwt or SubABmt. Total cell lysate samples were analyzed by IP with anti-caspase-1 antibody. Hc in (F) and (G) indicates the heavy chain of anti-caspase-1 IgG. Data are means ± SD (n = 3). ∗p< 0.05; ∗∗p< 0.01; ND, not detected. See also Figure S3.

During Gram-negative bacterial infection, LPS translocates to the cytoplasm by lysis of pathogen-containing vacuoles (Meunier et al., 2014) or bacterial outer membrane vesicles (Vanaja et al., 2016). Previous studies demonstrated that caspase-11 is an intracellular LPS sensor that is activated after cytoplasmic delivery of LPS (Shi et al., 2014) and is required for non-canonical inflammasome activation, caspase-1 activation, and IL-1β/IL-18 maturation in macrophages (Kayagaki et al., 2011). As seen in Figure 1, SubABwt inhibited caspase-11 expression, which may be associated with SubAB-mediated reduction of IL-1β production. To test this possibility, we suppressed expression of caspase-11 in J774.1 cells by using siRNA (Figure 3A). Intracellular LPS TF to LPS-primed J774.1 cells induced IL-1β release into the culture supernatant (Figures 3B and S3A). Caspase-11 knockdown attenuated this non-canonical inflammasome-dependent IL-1β release (Figure 3B). Treatment with SubABwt at 3 h after LPS priming inhibited LPS TF-induced IL-1β release, but SubABmt treatment produced no inhibition (Figure 3C). In this LPS TF model, activated caspase-11 was detected in the supernatant, but its level was reduced in the presence of SubABwt (Figure 3D). SubABwt also suppressed LPS TF-induced intracellular expression of pro-caspase-11 (Figure 3E). These data thus indicate that caspase-11 is critical for non-canonical inflammasome activation to promote IL-1β maturation. Our data also suggest that SubAB prevents non-canonical NLRP3 inflammasome activation by reducing pro-caspase-11 expression.

### SubAB impairs assembly of the NLRP3 inflammasome complex

To investigate the effect of SubAB on NLRP3 inflammasome activation, we next analyzed formation of the NLRP3 inflammasome complex in J774.1 cells. Immunoprecipitation with anti-caspase-1 antibody showed a successful pull-down of the NLRP3/pro-caspase-1 assembly in the lysate of STEC O113 WT-infected cells (Figure 3F). STEC O113 Δ*subAB* infection clearly enhanced formation of the NLRP3/pro-caspase-1 assembly but SubABwt treatment did not. Figure 3G clearly shows induction of the formation of the NLRP3/caspase-1 assembly in LPS-transfected cells, which was inhibited by SubABwt. STEC O113 Δ*subAB*-induced formation of the NLRP3/pro-caspase-1 assembly was downregulated in caspse-11 knockdown cells (Figure S3B). These results suggest that NLRP3 forms an inflammasome complex with caspase-1 in STEC O113 infection via caspase-11 activation, whereas SubAB inhibits caspase-11 expression and the interaction between NLRP3 and caspase-1.

### SubAB reduces LPS-induced IFN-β production by the IRE1α-dependent pathway

**Figure 4:**
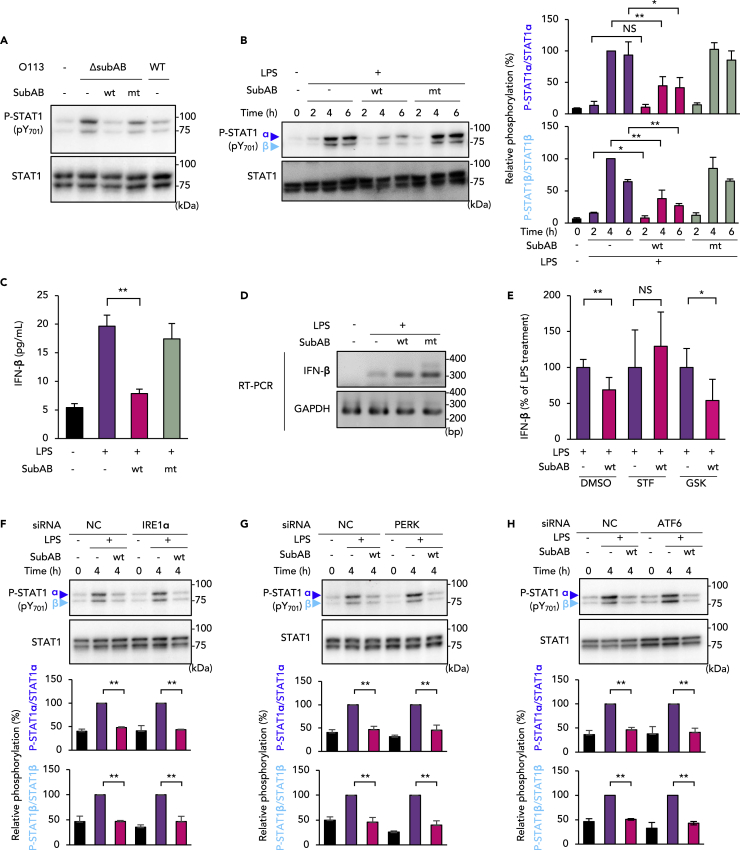
SubAB inhibits STAT1 phosphorylation through IRE1α-mediated attenuation of IFN-β production in J774.1 cells (A) J774.1 cells were infected with STEC O113 WT and STEC O113 Δ*subAB* for 6 h in the presence or absence of SubABwt or SubABmt. Phosphorylation of STAT1 (P-STAT1) α and β was analyzed by using WB with anti-P-STAT1 (pY701) and total STAT1 antibodies. (B) Cells were treated with LPS as indicated and analyzed by using WB with anti-P-STAT1 and total STAT1 antibodies. Relative P-STAT1 α and β amounts were quantified by densitometry (right panel). (C) Cells were treated with LPS for 6 h as indicated. IFN-β production in culture supernatants was analyzed by means of ELISA. (D) Cells were treated with LPS for 4 h with or without SubABwt or SubABmt. Total RNA extracted from the cells was subjected to RT-PCR with IFN-β and GAPDH primers. (E) Cells were pre-treated with 100 μM STF or 1 μM GSK for 1 h and were then treated with LPS for 6 h in the presence or absence of SubABwt or SubABmt. IFN-β production in culture supernatants was analyzed by using ELISA. (F–H) Cells were transfected with negative control (NC), IRE1α (F), PERK (G), or ATF6 (H) siRNA. After 72 h, cells were stimulated with LPS as indicated, and total cell lysate samples were analyzed by using WB with anti-P-STAT1 (pY701) and total STAT1 antibodies. Relative P-STAT1 α and β amounts were quantified by densitometry (lower panels). Data are means ± SD (n = 3). ∗p< 0.05; ∗∗p< 0.01; NS, not significant. See also Figure S3.

In macrophages, LPS-induced caspase-11 expression is regulated by transcription factors including signal transducer and activator of transcription 1 (STAT1). Toll-like receptor 4/TRIF-dependent production of the type I IFNs—IFN-α and IFN-β—stimulates STAT1 phosphorylation through receptor-associated Janus-activated kinases (Rathinam et al., 2012). We studied the effect of SubAB on STAT1 phosphorylation in J774.1 cells during STEC O113 infection. In STEC O113 Δ*subAB*-infected cells, STAT1 phosphorylation markedly increased at 6 h after infection (Figure 4A). Phosphorylation was suppressed in cells incubated with STEC O113 WT as well as STEC O113 Δ*subAB* plus SubABwt. As Figure 4B shows, STAT1 phosphorylation was fully induced by LPS treatment within 6 h, whereas SubABwt significantly inhibited STAT1 phosphorylation. Because STAT1 phosphorylation is initiated by IFN-β autocrine/paracrine stimulation, we next investigated the effect of SubAB on LPS-induced IFN-β production in J774.1 cells. Treatment with SubABwt significantly inhibited this LPS-induced IFN-β production (Figure 4C). We next analyzed LPS-induced IFN-β mRNA expression in the presence or absence of SubAB. Expression of LPS-induced IFN-β mRNA was enhanced rather than inhibited by SubABwt and SubABmt (Figure 4D). To determine which ER sensor protein is responsible for the inhibition of IFN-β production, we used the PERK inhibitor GSK and the IRE1α inhibitor STF and analyzed IFN-β protein production by ELISA. Figure 4E shows reduced IFN-β production in the presence of SubABwt. Treatment with STF or IRE1α knockdown negated the SubABwt-mediated reduction of IFN-β production (Figures 4E and S3C). Treatment with GSK or PERK knockdown, however, did not affect IFN-β production. We then investigated whether IRE1α was involved in SubAB-mediated inhibition of STAT1 phosphorylation. Contrary to our expectations, SubABwt still inhibited LPS-induced STAT1 phosphorylation even in IRE1α knockdown cells, as in PERK or ATF6 knockdown cells (Figures 4F and 4H). These results raised the possibility that SubAB inhibition occurs both upstream of IFN-β production via IRE1α and downstream of IFN-β via other ER stress sensors. Therefore, we next studied the effects of SubAB on IFN-β-induced STAT1 phosphorylation in J774.1 cells.

### SubAB blocks IFN-β-induced STAT1 phosphorylation via the PERK-dependent pathway

**Figure 5:**
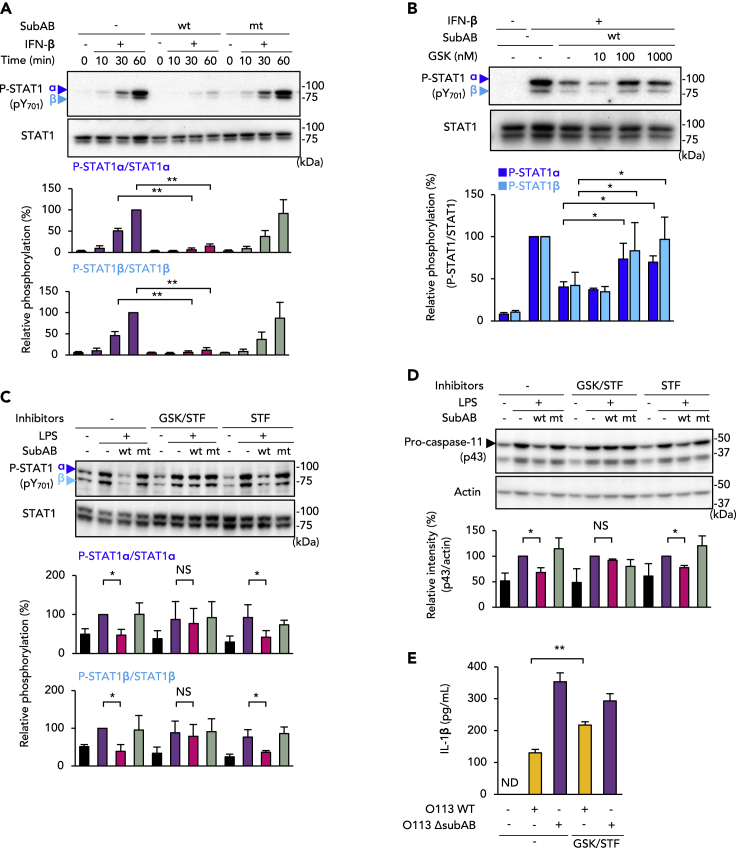
SubAB inhibits caspase-11 expression through PERK-mediated attenuation of IFN-β-induced STAT1 phosphorylation in J774.1 cells (A) Cells were pre-treated with SubABwt or SubABmt for 3 h or were not treated and were then stimulated with IFN-β (100 pg/mL) for the indicated times or were not so stimulated. (B) Cells were pre-treated with or without SubABwt. After 2 h, cells were treated with GSK for 1 h at one of the indicated concentrations, followed by treatment with IFN-β for 1 h. (C) Cells were treated with 1 μM GSK or 100 μM STF for 1 h, followed by stimulation with LPS (100 ng/mL) for 4 h with or without SubABwt or SubABmt. Total cell lysate samples were analyzed by using WB with anti-P-STAT1 (pY701) and total STAT1 antibodies (A–C). Relative P-STAT1 α and β levels were quantified by using densitometry (A–C, lower panels). (D) WB with anti-caspase-11 antibody for the cells in (C). The lower panel shows densitometric analysis for p43 band intensity. Actin served as the loading control. (E) Cells were treated with 1 μM GSK or 100 μM STF for 1 h, followed by infection with STEC O113 WT or STEC O113 Δ*subAB* as indicated. After 16 h, culture supernatants were subjected to ELISA for IL-1β. Data are means ± SD (n = 3). ∗p< 0.05; ∗∗p< 0.01; NS, not significant; ND, not detected. See also Figure S4.

IFN-β binds to IFN-α receptor (IFNAR), i.e., IFNAR1 and IFNAR2, in an autocrine and paracrine manner, which leads to STAT1 phosphorylation and caspase-11 expression (Rathinam et al., 2012). As mentioned above, knockdown of any ER stress sensors did not restore the inhibition of LPS-induced STAT1 phosphorylation by SubABwt (Figures 4F and 4H). We, therefore, studied whether SubAB would block downstream of IFN-β binding to IFNAR by determining the effect of SubAB on IFN-β-activated STAT1 phosphorylation in J774.1 cells. Treatment with IFN-β increased STAT1 phosphorylation in control and SubABmt-treated cells, whereas phosphorylation was significantly inhibited in SubABwt-treated cells (Figure 5A). In PERK knockdown cells, the inhibition of IFN-β-induced STAT1 phosphorylation by SubABwt was somewhat negated (Figure S4A). In IRE1α and ATF6 knockdown cells, SubABwt inhibited IFN-β-induced STAT1 phosphorylation, similar to the inhibition seen in negative control cells (Figures S4B and S4C). The PERK inhibitor GSK nullified the inhibition of STAT1 phosphorylation by SubABwt in a dose-dependent manner (Figure 5B). To determine whether IRE1α and PERK regulated LPS-induced STAT1 phosphorylation and caspase-11 expression, cells were treated with both GSK and STF before stimulation with LPS in the presence or absence of SubABwt. Inhibition of IRE1α and PERK by inhibitors abolished the SubABwt-mediated inhibition of STAT1 phosphorylation (Figure 5C) and caspase-11 expression (Figure 5D). In IRE1α knockdown cells, GSK reduced the inhibition of LPS-induced STAT1 phosphorylation by SubABwt (Figure S4D). In addition, as expected, in IRE1α knockdown cells GSK abolished the inhibitory effect of SubABwt on STEC O113 Δ*subAB* infection-induced IL-1β release (Figure S4E). Inhibition of IRE1α and PERK by inhibitors counteracted SubAB-mediated inhibition of STEC O113 Δ*subAB* infection-induced IL-1β release (Figure 5E). The ER stress inducers Tm and TG also inhibited both LPS- and IFN-β-induced STAT1 phosphorylation (Figures S4F and S4G). In particular, TG had inhibitory effects on IFN-β-induced STAT1 phosphorylation, which suggests that TG-mediated ER stress appeared to occur via the PERK-dependent pathway rather than the IRE1α-dependent pathway (Figure S4G). All these results together indicate that SubAB inhibits the caspase-11-mediated non-canonical inflammasome through activation of the IRE1α and PERK pathways, which leads to inhibition of IFN-β production and STAT1 phosphorylation, respectively (Figure S4H).

### SubAB inhibits intestinal caspase-1 activation and production of IL-1β and IL-18 and promotes intestinal survival of *C. rodentium*

**Figure 6:**
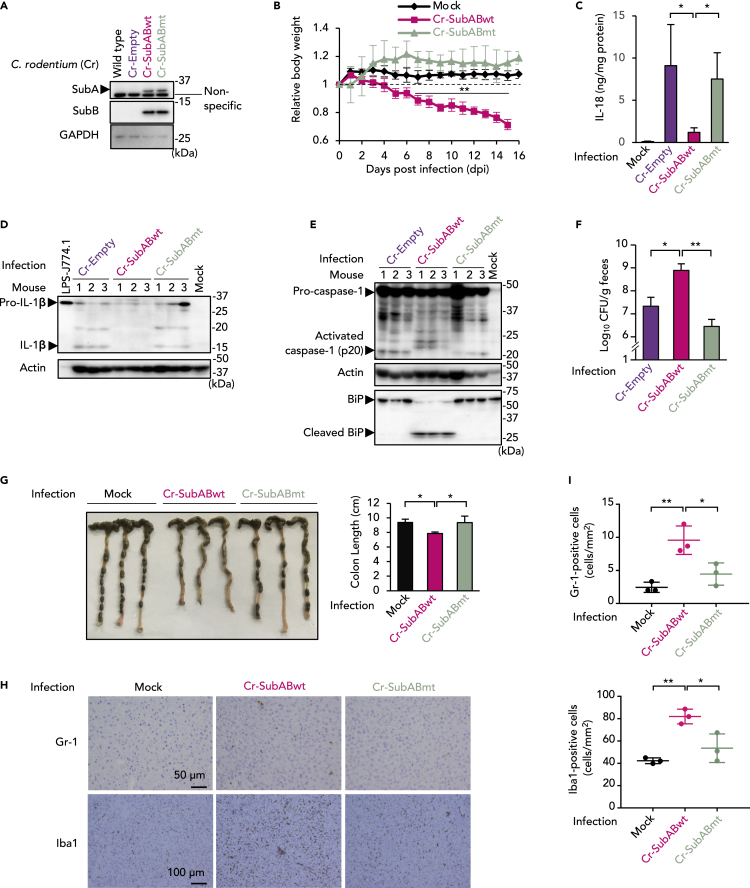
SubAB inhibits caspase-1 activation and production of IL-1β and IL-18 *in vivo* and promotes intestinal survival of ***C****. rodentium* (A) Preparation of SubAB-expressing *C. rodentium* (Cr-SubAB). WB image for expression of SubA (upper panel) and SubB (lower panel) in *C. rodentium* strains. GAPDH served as the loading control. (B) C57BL/6 mice received oral administration of Cr-SubABwt or Cr-SubABmt as described in the STAR Methods. Body weights of groups of five mice that received PBS (Mock), Cr-SubABwt, or Cr-SubABmt, expressed as relative changes from 0 days post infection (dpi). Data are averages (±SD) of 5 mice per group. (C) Tissue homogenates of intestines from infected mice at 11 dpi were subjected to ELISA for IL-18. Data are means ± SD (n = 3 or 4). (D) Tissue homogenates of intestines from infected mice were analyzed at 11 dpi by using WB with anti-IL-1β antibody. LPS-treated J774.1 cell lysate was used as a positive control for pro-IL-1β. (E) Tissue homogenates of intestines from infected mice were analyzed at 11 dpi using WB with anti-caspase-1 and anti-BiP antibodies. Actin (D and E) served as the loading control. (F) Mouse feces were collected at 11 dpi from intestines from three mice (three feces per mouse). The number of viable bacteria was determined by CFU analysis of plates; results are given as log_10_ CFU per gram of feces. Data are means ± SD (n = 9 per group). (G) Representative gross colon images from mice that received PBS (Mock), Cr-SubABwt, or Cr-SubABmt at 15–16 dpi. The right panel shows quantitative results of colon length for the image on the left. Data are means ± SD (n = 3 per group). (H) Immunostained micrographs of neutrophils (Gr-1, upper) and macrophages (Iba1, lower) in liver sections from mice in each group at 15–16 dpi. (I) The quantitative results of the infiltration in images from (H). Data are means ± SD (n = 3). ∗p< 0.05; ∗∗p< 0.01. See also Figures S5 and S6.

Development of a suitable animal infection model may help understanding of the contribution of SubAB to enteropathogenic bacterial infection. In fact, we describe here a murine infection model that we developed by constructing of SubAB-producing *C. rodentium*, as a model for the natural murine infection with enteropathogenic *E. coli* or STEC. We first electroporated SubAB-expression plasmids encoding an ampicillin (Amp)-resistance gene (pET23b-SubABwt, pET23b-SubABmt) into *C. rodentium* (Cr) to generate strains expressing SubABwt (Cr-SubABwt) or SubABmt (Cr-SubABmt). As Figure 6A illustrates, expression of SubA and SubB was detected in both Cr-SubABwt and Cr-SubABmt strains. Growth rates of these strains were the same under static conditions (Figure S5A) and shaking conditions (Figure S5B). Figure S5C shows definite cleavage of BiP in Cr-SubABwt-infected J774.1 cells, which indicates that the Cr transformants expressed active SubAB. We then treated C57BL/6 mice with PBS (Mock) or infected them with Cr-SubABwt or Cr-SubABmt via oral gavage and monitored their body weight change. Mock-treated and Cr-SubABmt-infected mice maintained body weight throughout the infection period. In contrast, mice infected with Cr-SubABwt lost more than 10% of their initial weight by 7 days post infection (dpi) and more than 25% by 15 dpi (Figures 6B and S5D). At 4, 6, and 11 dpi, we analyzed BiP cleavage as an indicator of SubABwt production in the intestine of infected mice (Figure S5E). Cleaved BiP was clearly detected in intestinal homogenates of Cr-SubABwt-infected mice. Cleavage of BiP became stronger in a time-dependent manner, which indicates that Cr-SubABwt-derived SubABwt increased in the mouse intestine until 11 dpi.

Because SubAB suppressed production of IL-1β and IL-18 through inhibition of inflammasome activation in J774.1 cells, we investigated production of IL-1β and IL-18 and inflammasome activation in the mouse model. Cr-Empty- and Cr-SubABmt-infected mice produced IL-18 in the intestine at 11 dpi; however, Cr-SubABwt-infected mice had markedly reduced IL-18 production (Figure 6C). As Figure 6D shows, the intestinal pro-IL-1β expression was induced in all mice after infection with Cr strains. Maturation of IL-1β occurred in Cr-Empty- and Cr-SubABmt-infected mice but not in Cr-SubABwt-infected mice (Figure 6D). Inasmuch as inflammasome activation was responsible for proteolytic maturation and secretion of bioactive IL-1β and IL-18 (Martinon et al., 2002), we then investigated the effects of SubAB on caspase-1 activation in the colon of infected mice. We found activated caspase-1 (p20) in the intestine of Cr-Empty-infected and Cr-SubABmt-infected mice but not Cr-SubABwt-infected mice (Figure 6E). In agreement with results for J774.1 cells, SubABwt attenuated the infection-induced expression of intestinal caspase-11 (Figure S5F).

Previous studies showed that IL-18, NLRP3, or caspase-1 was critical for host defense and that their deficiency enhanced susceptibility to *C. rodentium* (Liu et al., 2012). We evaluated the effects of SubAB-induced inhibition of IL-1β/IL-18 on intestinal *C. rodentium* survival by determining the number of viable bacteria in feces. At 11 dpi, bacterial CFUs for feces from Cr-SubABwt-infected mice group were significantly higher than CFUs for the Cr-Empty- and Cr-SubABmt-infected groups (Figure 6F). A similar tendency was observed at 6 dpi for Cr-SubABwt-infected mice relative to Cr-Empty- and Cr-SubABmt-infected mice; the difference was not statistically significant, however (Figure S5G). We then confirmed the identity of feces-derived colonies as Cr-SubABwt by analyzing whether they could induce BiP cleavage during infection of J774.1 cells. Inocula obtained from mouse feces infected with Cr-SubABwt induced BiP cleavage in J774.1 cells, which indicated that Cr-SubABwt formed isolated colonies that expressed active SubAB (Figure S5H). These results suggest that SubAB may promote *C. rodentium* proliferation in mouse colon by disturbing colonic host defense responses through inhibiting inflammasome activation as well as IL-1β/IL-18 production.

### SubAB exacerbated colon damage caused by *C. rodentium* infection

To evaluate the enhanced susceptibility to and systemic symptoms caused by *C. rodentium* in Cr-SubABwt-infected mice, we examined the gross intestinal anatomy and liver and spleen histology of mice that were used in the weight monitoring and survival assay. Consistent with the weight loss illustrated in Figures 6B and S5D, we found intestinal hyperplasia with reduced colon length and diarrhea in Cr-SubABwt-infected mice (Figure 6G). Intestinal hyperplasia is a typical pathological feature of *C. rodentium* infection; it is characterized by thickening of the colonic mucosa caused by excessive regeneration of epithelial cells (Collins et al., 2014). Our results suggested that the hyperplasia and diarrhea were caused by an increased Cr-SubABwt bacterial load in the intestine. We then studied the pathology of systemic inflammation in livers and spleens from infected mice. To study the infiltration of macrophages and neutrophils, we stained liver sections with the macrophage/microglial marker Iba1 or neutrophil marker Gr-1. Consistent with the enhanced symptoms, infiltration of macrophages and neutrophils in the liver of Cr-SubABwt-infected mice was significantly higher than infiltration in other mice (Figures 6H and 6I). Spleen enlargement occurs in various diseases, including bacterial infections. In both Cr-SubABwt- and Cr-SubABmt-infected mice, spleen weights were higher than those of mock-treated mice (Figure S6A). These results suggest that SubAB inhibits intestinal inflammasome-mediated cytokines and thereby exacerbates the enteropathogenic bacterial infection, although SubAB does not inhibit systemic inflammation outside the colon. In summary, our findings indicate that SubAB may be associated with STEC survival and promotion of the infectious disease (Figure S6B).

## Discussion

SubAB induces various host cell responses such as induction of cell death and stress granule formation, inhibition of protein synthesis, suppression of iNOS expression, and impairment of autophagy (Tsutsuki et al., 2020). We demonstrated here that SubAB strongly inhibited host defense responses to *C. rodentium* infection, at least in part by suppressing inflammasome activation. Our *in vitro* experiments revealed that SubAB can interfere with non-canonical inflammasome activation by triggering PERK- and IRE1α-dependent ER stress signaling responses (Figure S4H). Upon mammalian ER stress, IRE1α suppresses protein translation via regulated IRE1-dependent decay, a degradation of ER-localized mRNAs through its RNase domain. Several substrates for IRE1α RNase with a consensus mRNA sequence similar to X-box binding protein 1 splicing sites were reported (Maurel et al., 2014). SubAB induced IRE1-dependent decay in B cells, which led to reduced antibody production (Tang et al., 2018). PERK, however, suppressed protein translation via phosphorylation of the eukaryotic initiation factor 2α (eIF2α)-dependent or-independent pathway (Guan et al., 2014; Harding et al., 2000). SubAB transiently inhibited protein translation and induced cell cycle arrest in parallel with phosphorylation of PERK and eIF2α (Morinaga et al., 2008), which suggests that SubAB-induced ER stress inhibits pro-IL-1β protein translation via the PERK-dependent pathway. SubAB induced phosphorylation of eIF2α and stress granule formation through the PERK-dependent pathway (Tsutsuki et al., 2016). Under conditions of stress, stress granules participate in the arrest of mRNA translation, but the role of SubAB-induced stress granule formation during STEC infection has not yet been determined. Moreover, emerging evidence highlights the roles of RNA-binding proteins including Regnase-1, Roquin (Mino et al., 2015), and Arid5a (Masuda et al., 2013) in the regulation of cytokine expression by mRNA degradation or stabilization. Thus, our findings suggest that SubAB-mediated pro-IL-1β and IFN-β inhibition may require crosstalk between IRE1α- and PERK-mediated signaling.

Menu et al. reported that chemical ER stress inducers activated the NLRP3 inflammasome in LPS-primed macrophages (Menu et al., 2012). In this mechanism, unfolded protein responses via PERK, IRE1α, and ATF6 were not involved in inflammasome activation. In addition, another report indicated that chemical ER stress inducers activated the NLRP3 inflammasome depending on production of ROS as well as caspase-2 (Bronner et al., 2015). Canonical NLRP3 inflammasome activation is thought to be a two-step process. An initial priming signal induces expression of NLRP3 and pro-IL-1β/IL-18 through NF-κB activation; the activation signal is provided by various stimuli including bacterial pore-forming toxins, extracellular ATP, and particulate matter (He et al., 2016). These studies suggest that ER stress acts as an activation signal in canonical inflammasome activation. We investigated STEC-induced non-canonical inflammasome activation, unlike these canonical models, and found inhibitory effects of SubAB. Our results therefore indicate an essential role of ER stress on the non-canonical inflammasome during bacterial infection.

LPS-induced pro-caspase-11 expression requires STAT1 phosphorylation via IFN-β stimulation (Rathinam et al., 2012). We investigated SubAB-mediated inhibition mechanisms of the LPS/IFN-β/STAT1 phosphorylation pathway as an upstream signal of pro-caspase-11 expression. We found inhibitory effects of SubAB-activated IRE1α and PERK, upstream and downstream of IFN-β production (Figures 4, 5, and S4), respectively. IRE1α and PERK simultaneously blocked the two consecutive processes in STAT1 phosphorylation, so SubAB produced potent disruption of LPS-induced STAT1 phosphorylation and caspase-11 expression. ER stress is correlated with internalization or degradation of IFNAR1 and the reduced surface expression of IFNAR1 (Gunduz et al., 2012; Liu et al., 2009). Our results suggested that SubAB may inhibit receptor binding with IFN-β via reduced surface expression of IFNAR1. We propose that future studies should be performed to determine whether SubAB perturbs the cell surface expression of IFNAR1. The evidence so far, together with our findings, suggests that application of inhibitors of the PERK and IRE1α pathways may become a therapeutic tool for ER stress-related bacterial infection.

To date, no *in vivo* studies have indicated that SubAB inhibits host innate immunity and enhances survival of enteropathogenic *E. coli*. Mallick et al. developed a murine infection model for STEC by constructing Stx-producing *C. rodentium*, followed by oral gavage in C57BL/6 mice (Mallick et al., 2012). Their study showed that Stx induced weight loss, severe disease warranting euthanasia, and intestinal tissue damage in mice that received Stx-producing *C. rodentium*. Stx did not promote intestinal bacterial growth, however. A recent study also developed a mouse infection model that involved oral administration of Stx-producing *C. rodentium*, and it demonstrated that Stx did not promote intestinal bacterial growth (Havira et al., 2020). These findings suggest that Stx is not required for bacterial growth in mouse intestine. In addition, Stx suppressed the inflammasome response in mouse macrophages and the *in vivo* model. In a human model, however, Stx activated the inflammasome by co-transport with LPS in PMA-differentiated THP-1 macrophages via binding with the Stx-receptor globotriaosylceramide (GB3 or CD77) (Platnich et al., 2018). Murine macrophages lack surface expression of CD77, which may lead to the opposite involvement of Stx in the inflammasome response between humans and mice. In our studies here, we developed an infection model for an SubAB-producing intestinal pathogen by using *C. rodentium* carrying the SubAB-expression plasmid (Figure 6). Consistent with previous models, mice infected with vector control Cr-Empty or inactive SubAB-producing Cr-SubABmt did not lose weight. Unlike Stx, SubAB caused increased recovery of live Cr-SubABwt organisms in feces from mouse intestine (Figure 6F). Thus, intrinsic pathogenic effects of *C. rodentium* including weight loss (Figure 6B), intestinal hyperplasia, shortened colon length, and diarrhea (Figure 6G) were elicited, which suggests that SubAB exacerbates the symptoms of *C. rodentium* infection. Besides these symptoms, obvious tissue injury and gastrointestinal hemorrhage were not observed under the present experimental conditions. This finding may be because of less translocation of SubAB into blood vessels. Consistent with results from our *in vitro* study, SubAB inhibited IL-1β/IL-18 production, caspase-1 activation, and caspase-11 expression *in vivo* (Figures 6C–6E and S5F). Because of IL-1β/IL-18 production via non-canonical inflammasome activation is critical to control *C. rodentium* infection (Liu et al., 2012), our data suggest that SubAB may promote survival of *C. rodentium* in the intestine via IL-1β/IL-18 inhibition. In addition, our *in vivo* experiment demonstrated that SubAB did not suppress several symptoms including colonic hyperplasia, infiltration of macrophages and neutrophils into the liver, and spleen hypertrophy (Figures 6G–6I and S6A).*C. rodentium*-induced colonic hyperplasia was observed even in caspase-1-deficient or IL-18-deficient mice (Liu et al., 2012). These observations suggest that IL-1β and IL-18 are not responsible for *C. rodentium*-induced intestinal hyperplasia and that SubAB does not inhibit systemic inflammation except for intestinal inflammation. Wang et al. injected recombinant SubAB intraperitoneally into mice and found splenic atrophy as a result (Wang et al., 2007). In our infection-based study, we saw no reduced spleen size. These data may be caused by differences in dosing methods, injection routes or toxin levels of the SubAB injected, or the presence or absence of infection. Therefore, our *in vivo* data suggest that SubAB impairs the inflammasome in the host intestine and exacerbates symptoms of enteropathogenic bacterial infection.

Caspase-11 acts as an intracellular LPS sensor in Gram-negative bacterial infection (Shi et al., 2014) and regulates non-canonical inflammasome activation (Kayagaki et al., 2011). Non-cytosolic bacteria-derived LPS is delivered by bacterial outer membrane vesicles into host cytosol (Vanaja et al., 2016). Cytosolic bacteria also release LPS into the cytosol after lysis by guanylate-binding protein (Meunier et al., 2014). To confirm whether SubAB could prevent intracellular LPS-mediated caspase-11 activation, we utilized an LPS TF model (Figure S3A). LPS TF induced formation of the caspase-1 and NLRP3 assembly, which was attenuated by SubAB (Figure 3G). Caspase-11 was necessary to activate the STEC-induced NLRP3 inflammasome (Figure S3B), which suggests that SubAB-mediated caspase-11 suppression disrupted NLRP3 inflammasome activation.

Intracellular LPS and inflammasome-dependent cytokines have important antimicrobial functions (Vanaja et al., 2015). Several pathogenic bacteria can escape from a host defense system by disrupting inflammasome activation via virulence factors. The *Shigella flexneri* OspC3 effector attenuates activity of caspase-4 (human ortholog of mouse caspase-11) by preventing heterodimerization between caspase-4-p19 and caspase-4-p10 (Kobayashi et al., 2013). Enteropathogenic *E. coli* bacteria produce the virulence factor NleF, which targets the catalytic domain of caspase-4 and inhibits its proteolytic activity (Pallett et al., 2017). Given the importance of caspase-4 and caspase-11 in inflammasome activation, SubAB may act as an anti-inflammasome factor to enhance STEC survival and worsen Stx-mediated infectious diseases (Figure S6B). All our current data thus clearly indicate the critical importance of SubAB on STEC survival mechanisms *in vivo*, in addition to reported mechanisms, such as inhibition of the production of antimicrobial substances including IgM (Hu et al., 2009), NO (Tsutsuki et al., 2012), and lipocalin-2 (Yahiro et al., 2020). Finally, we believe that our research on pathogenic enterotoxins contributes to the establishment of a therapeutic strategy to overcome bacterial infection and development of tools to study the inflammasome.

### Limitations of the study

Our study showed that SubAB impaired non-canonical inflammasome activation and exacerbated bacterial infection. We provided certain clues, such as the ER stress sensor, involved in inhibition of the inflammasome, but the detailed downstream mechanism remains unclear. In particular, details of how ER stress downregulates the IFN-β/STAT1 pathway are undefined. We developed a murine infection model by using *C. rodentium* carrying a SubAB-expression plasmid, but investigating the effect by using the STEC O113 infection model in the future is necessary. We investigated the inflammasome-suppressing effect of SubAB by using cultured mouse cells, but the effect on the inflammasome of human cells was not clarified. Previous reports suggested that the effect of Stx on inflammasome activation may differ between human and mouse cells; therefore, additional studies are needed to better explore the relationship between SubAB and human cells during inflammasome activation.

## STAR★Methods

### Key resources table

**Table:** 

REAGENT or RESOURCE	SOURCE	IDENTIFIER
**Antibodies**		

Rabbit polyclonal anti-actin	Sigma-Aldrich	Cat#A2066; RRID: AB_476693
Mouse monoclonal anti-NLRP3	Cell Signaling Technology	Cat#15101; RRID: AB_2722591
Rabbit monoclonal anti-phospho-STAT1	Cell Signaling Technology	Cat#7649; RRID: AB_11220426
Rabbit polyclonal anti-STAT1	Cell Signaling Technology	Cat#9172; RRID: AB_10693929
Rabbit monoclonal anti-PERK	Cell Signaling Technology	Cat#3192; RRID: AB_2095847
Rabbit monoclonal anti-IRE1α	Cell Signaling Technology	Cat#3294; RRID: AB_823545
Rabbit monoclonal anti-caspase-11 (clone EPR18628)	Abcam	Cat#ab180673; RRID: N/A
Rabbit monoclonal anti-GSDMD (clone EPR19828)	Abcam	Cat#ab209845; RRID: AB_2783550
Rabbit polyclonal anti-GAPDH	GeneTex	Cat#GTX100118; RRID: AB_1080976
Rat monoclonal anti-caspase-11(9D11)	BioLegend	Cat#647201; RRID: AB_1937283
Mouse monoclonal anti-BiP/Grp78	BD Biosciences	Cat#610978; RRID: AB_398291
Mouse monoclonal anti-caspase-1 p20 (clone Casper-1)	AdipoGen	Cat#AG-20B-0042-C100; RRID: AB_2755041
Mouse monoclonal anti-ATF6 (clone 37-1)	BioAcademia	Cat#BAM-73-505-EX; RRID: AB_10709801
Goat polyclonal anti-IL-1β	R&D Systems	Cat#AF-401-NA; RRID: AB_416684
Rabbit polyclonal SubAB	(Yahiro et al., 2006)	N/A
Rabbit polyclonal anti-Iba1	Fujifilm Wako Pure Chemical Corp	Cat#019–19741; RRID: AB_839504
Rat monoclonal anti-Gr-1	SouthernBiotech	Cat#1900-01; RRID: AB_2795462
Anti-mouse IgG, HRP-linked antibody	Cell Signaling Technology	Cat#7076; RRID: AB_330924
Anti-rabbit IgG, HRP-linked antibody	Cell Signaling Technology	Cat#7074; RRID: AB_2099233
Anti-mouse IgG Veriblot for IP secondary antibody (HRP) ab131368	Abcam	Cat#ab131368; RRID: AB_2895114
HRP-conjugated anti-goat secondary antibody	R&D Systems	Cat#HAF017; RRID: AB_562588
HRP-conjugated anti-mouse secondary antibody	GE Healthcare	Cat#NA931; RRID: AB_772210
HRP-conjugated anti-rabbit secondary antibody	GE Healthcare	Cat#NA934; RRID: AB_772206
HRP-conjugated anti-rat secondary antibody	GE Healthcare	Cat#NA9350, RRID: AB_772192

**Bacterial and viral strains**		

STEC O113:H21 WT	(Tsutsuki et al., 2016)	N/A
STEC O113:H21 Δ*subAB*	(Tsutsuki et al., 2016)	N/A
*Escherichia coli* BL21(DE3)	(Yahiro et al., 2006)	N/A
*Citrobacter rodentium*	ATCC	ATCC 51459

**Chemicals, peptides, and recombinant proteins**		

Recombinant His-tagged SubABwt	(Morinaga et al., 2007)	N/A
Recombinant His-tagged SubABmt	(Morinaga et al., 2007)	N/A
LPS (*Escherichia coli* O55:B5)	Sigma-Aldrich	Cat#L2880
STF-083010	Abcam	Cat#ab146176; CAS: 307543-71-1
GSK2656157	Calbiochem	Cat#504651; CAS: 1337532-29-2
IFN-β	R&D Systems	Cat#8234-MB-010
Thapsigargin	Fujifilm Wako Pure Chemical Corp.	Cat#209-17281; CAS: 67526-95-8
Tunicamycin	Fujifilm Wako Pure Chemical Corp.	Cat#202-08241; CAS: 11089-65-9
Dulbecco’s Modified Eagle’s medium (DMEM)	Fujifilm Wako Pure Chemical Corp	Cat#044-29765
Opti-MEM	Thermo Fisher Scientific	Cat#51985-034
Penicillin-Streptomycin Mixed Solution	Nacalai Tesque	Cat#26253-84
Fetal Bovine Serum, CELLect Gold, US Origin	MP Biomedicals Inc	Cat#2916754
Lipofectamine RNAiMAX Transfection Reagent	Thermo Fisher Scientific	Cat#13778075
Lipofectamine 2000 Transfection Reagent	Thermo Fisher Scientific	Cat#11668027
Immobilon-P PVDF membrane	Merck Millipore	Cat#IPVH00010
Protease inhibitor cocktail set I, Animal-derived free (for general use) (x100)	Fujifilm Wako Pure Chemical Corp.	Cat#161-26023
Immobilon Western Chemiluminescent HRP Substrate	Merck Millipore	Cat#WBKLS0500
PrimeScript RT Master Mix	Takara Bio Inc.	Cat#RR036
TB Green Premix Ex Taq II	Takara Bio Inc.	Cat#RR820
ReverTra Ace qPCR kit	TOYOBO	Cat#FSQ-101
KOD FX	TOYOBO	Cat#KFX-101
Brain Heart Infusion (BHI) broth	Becton, Dickinson and Company	Cat#237500
Ni-NTA Agarose	Qiagen	Cat#30210
Recombinant Protein G Agarose	Thermo Fisher Scientific	Cat#15920010
Ampicillin sodium	Fujifilm Wako Pure Chemical Corp.	Cat#012-23303; CAS: 69-52-3
Gentamicin sulfate	Fujifilm Wako Pure Chemical Corp.	Cat#075-06451; CAS: 1405-41-0
cOmplete, Mini, EDTA-free Protease inhibitor cocktail	Roche	Cat#11836170001
MacConkey agar	Nissui Pharmaceutical Co., Ltd	Cat#05037
Difco LB Broth, Miller (Luria-Bertani)	Becton, Dickinson and Company	Cat#244620
Histofine Simple Stain Mouse MAX PO (R)	NICHIREI BIOSCIENCES	Cat#414341
Histofine Simple Stain MAX-PO (R)	NICHIREI BIOSCIENCES	Cat#424142
Histofine DAB substrate kit	NICHIREI BIOSCIENCES	Cat#425011

**Critical commercial assays**		

Quantikine® ELISA mouse IL-1β/IL-1F2	R&D Systems	Cat# SMLB00C
VeriKine^TM^ Mouse IFN-Beta ELISA Kit	PBL Assay Science	Cat#42400-1
Mouse IL-18 ELISA Kit	MBL	Cat#7625
RNeasy Mini Kit (250)	Qiagen	Cat#74106
Protein Assay BCA Kit	Fujifilm Wako Pure Chemical Corp	Cat#297-73101
Cytotoxicity LDH Assay Kit-WST	DOJINDO	Cat#CK12

**Experimental models: Cell lines**		

J774.1 cells (Female)	RIKEN BioResource Center	Cat#RCB0434

**Experimental models: Organisms/strains**		

C57BL/6J Mice (Female)	Japan SLC Inc	N/A

**Oligonucleotides**		

Primer for qPCR: mouse IL-1β forward: 5′-TGACGGACCCCAAAAGATG-3′	(Fujiwara et al., 2018)	N/A
Primer for qPCR: mouse IL-1β reverse: 5′-GCGAGATTTGAAGCTGGATG-3′	(Fujiwara et al., 2018)	N/A
Primer for qPCR: mouse GAPDH forward: 5′-TGCGACTTCAACAGCAACTC-3′	This study	N/A
Primer for qPCR: mouse GAPDH reverse: 5′-CCTGTTGCTGTAGCCGTATTC-3′	This study	N/A
Primer: mouse IFN-β forward: 5′-AAACAATTTCTCCAGCACTG-3′	(Zhang et al., 2019)	N/A
Primer: mouse IFN-β reverse: 5′-ATTCTGAGGCATCAACTGAC-3′	(Zhang et al., 2019)	N/A
Primer: mouse GAPDH forward: 5′-TGAGGCCGGTGCTGAGTATG-3′	(Furukawa et al., 2011)	N/A
Primer: mouse GAPDH reverse: 5′-CCTTCCACAATGCCAAAGTT-3′	(Furukawa et al., 2011)	N/A
siRNA targeting sequence: mouse PERK 5′-CACAAGCTGGGTCGCCATTTA-3′	Designed for mouse with reference to (Akazawa et al., 2013)	N/A
siRNA targeting sequence: mouse IRE1α 5′-GGATGTAAGTGACCGAATA-3′	Designed for mouse with reference to (Ruan et al., 2013)	N/A
siRNA targeting sequence: mouse ATF6 5′-CAGCTACCACCCACAACAA-3′	Designed for mouse with reference to (Shinkai et al., 2010)	N/A
siRNA: mouse caspase-11: siGENOME Mouse Casp4 (12363) siRNA	GE Healthcare Dharmacon	Cat#D-042432-01-0005

**Recombinant DNA**		

pET23b(+)	Novagen	Cat#69771
pET23b-SubABwt	(Morinaga et al., 2007)	N/A
pET23b-SubABmt	(Morinaga et al., 2007)	N/A

**Software**		

GraphPad Prism 7.0	GraphPad Software	https://www.graphpad.com
Image Lab Software for PC Version 6.0.1	BioRad	https://www.bio-rad.com/ja-jp/product/image-lab-software?ID=KRE6P5E8Z

### Resource availability

#### Lead contact

Further information and requests for reagents and resources should be directed to and will be fulfilled by the lead contact, Hiroyasu Tsutsuki (tsutsuki@kumamoto-u.ac.jp).

#### Materials availability

Recombinant His-tagged SubAB toxins generated in this study will be made available on request by the lead contact with a completed Materials Transfer Agreement.

### Experimental model and subjectdetails

#### Ethics statement

All animal experiment procedures were approved by the Kumamoto University Ethics Review Committee for Animal Experimentation and were performed with an effort to minimize the number of animals used and their suffering.

#### Bacterial strains

The LEE-negative but *stx2*- and *subAB*-positive *Escherichia coli* O113:H21 (STEC O113 WT) strain was clinically isolated from a patient with thrombotic thrombocytopenic purpura in Japan. To establish an O113 Δ*subAB* strain, the *subAB* gene in STEC O113:H21 was disrupted by the inserting a kanamycin resistance gene (*kan*), as previously described (Datsenko and Wanner, 2000) (Tsutsuki et al., 2016). These strains (O113 WT, O113 Δ*subAB*) were cultured in brain heart infusion (BHI) broth (Becton, Dickinson and Company, Franklin Lakes, NJ, USA) for 12 h at 37°C with shaking at 150 rpm. SubAB-expressing *E. coli* BL21(DE3) strains prepared as previously described (Yahiro et al., 2006) were cultured in Luria-Bertani (LB) broth (Becton, Dickinson and Company) supplemented with 100 μg/mL ampicillin for 12 h at 37°C with shaking at 150 rpm. The cultures were diluted 1:100 with fresh media and sub-cultured for 3–4 h (until the OD_600_ was 0.5–1.0).*Citrobacter rodentium* (*C. rodentium*) ATCC 51459 was purchased from AmericanType Culture Collection (ATCC) (Manassas, VA, USA) and was cultured in LB broth for 12 h at 37°C with shaking at 150 rpm. *C. rodentium* (Cr) strains producing SubABwt or SubABmt (Cr-SubABwt, Cr-SubABmt) were prepared by electroporation of the His-tag SubAB expression vector (pET23b-SubABwt, pET23b-SubABmt).*C. rodentium* electrocompetent cells were prepared by growing 50 mL of subculture in LB broth for 2–3 h at 37°C with shaking until the OD_600_ was 0.5–1.0. Cells were then pelleted by centrifugation and resuspended four times in 40 mL of sterile cold 10% glycerol, followed by a final resuspension in 1 mL of sterile cold 10% glycerol. pET23b (empty vector), pET23b-SubABwt, or pET23b-SubABmt (5 μg each) was added to 100 μL of the electrocompetent cells, and the cell/DNA mixture was placed into an ice-cold 1-mm gap electroporation cuvette (Nepa Gene, Chiba, Japan). Cells were electroporated at 1800 V, 25 μF, and 600 Ω by using a BioRad MicroPulser (BioRad, Hercules, CA, USA). Immediately after electroporation, cells were resuspended in 1 mL of LB, followed by plating 0.1 mL of the resuspension onto LB agar containing 100 μg/mL ampicillin. After incubation overnight at 37°C, ampicillin-resistant colonies were isolated and grown and SubAB expression was confirmed by using Western blotting (WB) with anti-SubAB antiserum. SubAB-producing Cr strains (Cr-SubABwt or Cr-SubABmt) or pET23b empty vector control cells (Cr-Empty) were treated with 0.2 mM isopropyl β-D-1-thiogalactopyranoside (IPTG) for 3 h before infection of mice as described below. For the bacterial growth assay, Cr strains were cultured overnight at 37°C in LB broth supplemented with 100 μg/mL ampicillin. Overnight cultures were then diluted 1,000-fold with fresh media. Diluted bacterial suspensions were cultured under static conditions for 6 h or under shaking conditions for 48 h at 37°C. Bacterial growth was determined by measuring the optical density at 595 nm with an iMark Microplate Reader (Bio-Rad) or BioSpectrometer kinetic spectrophotometer (Eppendorf, Hamburg, Germany). All strains were stored at −80°C in glycerol stock until used.

#### Cell culture

Cells of the mouse macrophage-like cell line J774.1 cells (RIKEN BioResource Center, Tsukuba, Japan) were cultured in Dulbecco’s Modified Eagle’s medium (DMEM) (Fujifilm Wako Pure Chemical Corp., Osaka, Japan) supplemented with 10% heat-inactivated fetal bovine serum (FBS) (CELLect Gold, US Origin; MP Biomedicals Inc., Solon, OH, USA), 100 U/mL penicillin, and 0.1 mg/mL streptomycin (Nacalai Tesque, Kyoto, Japan) in a 5% CO_2_ humidified incubator at 37°C.

#### Mouse model

Female 8-week-old C57BL/6J mice were purchased from Japan SLC Inc. (Shizuoka, Japan) and housed in the Center for Animal Resources and Development, Kumamoto University. Mice were maintained under a 12-h light/12-h dark cycle with free access to water and standard mouse diet.

### Method details

#### Reagents

Anti-actin rabbit polyclonal antibody and LPS O55:B5 were purchased from Sigma-Aldrich (St. Louis, MO, USA). Anti-PERK, anti-phospho-STAT1 (pY701), anti-STAT1, anti-NLRP3, anti-IRE1α antibodies, and horseradish peroxidase (HRP)-conjugated anti-rabbit and anti-mouse secondary antibodies were from Cell Signaling Technology (Danvers, MA, USA). Anti-caspase-11 rabbit monoclonal antibody, anti-mouse IgG VeriBlot for IP secondary antibody (HRP), and IRE1α inhibitor (STF-083010) were from Abcam (Cambridge, MA, USA). Anti-IL-1β goat polyclonal antibody, HRP-conjugated anti-goat secondary antibody, and recombinant mouse IFN-β were from R&D Systems (Minneapolis, MN, USA). HRP-conjugated anti-rabbit, anti-mouse, and anti-rat secondary antibodies were from GE Healthcare UK Ltd., (Buckinghamshire, England, UK). Anti-BiP/Grp78 mouse monoclonal antibody was from BD Biosciences (San Jose, CA, USA); anti-GAPDH rabbit polyclonal antibody was from GeneTex (Irvine, CA, USA); anti-ATF6 mouse monoclonal antibody was from BioAcademia (Osaka, Japan); anti-caspase-11 rat monoclonal antibody was from BioLegend (San Diego, CA, USA); anti-caspase-1 mouse monoclonal antibody was from AdipoGen (San Diego, CA, USA); PERK inhibitor II (GSK2656157) was from Calbiochem, Merck Millipore (Darmstadt, Germany). Rabbit polyclonal anti-Iba1 was from Fujifilm Wako Pure Chemical Corp. Rat monoclonal anti-Gr-1 was from SouthernBiotech (Birmingham, AL, USA). Anti-SubAB antiserum was prepared as reported previously (Yahiro et al., 2006).

#### Preparation of SubAB

Recombinant His-tagged wild-type SubAB (SubABwt) and catalytically inactive mutant SubA_S272A_B (SubABmt) were synthesized in *E. coli* BL21 (DE3) and purified by using Ni-NTA resin affinity chromatography as reported previously (Morinaga et al., 2007; Yahiro et al., 2006). SubABwt and SubABmt were used at 0.5 μg/mL for cell treatments in each experiment throughout this study.

#### Cell treatment and gene knockdown by inhibitors and siRNA transfection

J774.1 cells were seeded in 24, 48, or 96-well plates (at 2.5 × 10^5^, 1 × 10^5^, or 5 × 10^4^ cells per well, respectively). Cells were stimulated with LPS (100 ng/mL) or IFN-β (100 pg/mL) in the presence or absence of SubABwt or SubABmt (0.5 μg/mL) as indicated in the Figure Legends. To inhibit PERK or IRE1α, cells were pre-treated with GSK or STF for 1 h before LPS or IFN-β stimulation. Alternatively, RNA interference-mediated gene knockdown was performed with small-interfering RNAs (siRNAs) as previously described (Yahiro et al., 2012). siRNA for mouse PERK, mouse IRE1α, or mouse ATF6 was synthesized by Sigma-Aldrich Japan (Tokyo, Japan) (Akazawa et al., 2013; Ruan et al., 2013; Shinkai et al., 2010). Caspase-11 siRNA (siGENOME Mouse Casp4 siRNA) was purchased from GE Healthcare Dharmacon (Lafayette, CO, USA). J774.1 cells (2 × 10^4^ cells per well in 96-well plates; 1 × 10^5^ cells per well in 24-well plates) were transfected with the indicated siRNAs (at 100 nM) by using Lipofectamine RNAiMax transfection reagent (Thermo Fisher Scientific, Waltham, MA, USA) according to the manufacturer’s protocol. After 24 h, secondary transfection was performed just as for the primary transfection. At 72 h after transfection, cells were used in experiments. Transfection efficiency was evaluated by using WB and the indicated antibodies.

#### Macrophage infection

STEC O113 or Cr strains were subcultured in specified media, and the bacterial colony-forming units (CFU)/mL value was calculated via counting colonies on agar plates. For macrophage infection, J774.1 cells were cultured in antibiotic-free DMEM containing 10% heat-inactivated FBS and were infected as previously described (Tsutsuki et al., 2012). Briefly, J774.1 cells were seeded in 24, 48, or 96-well plates (at 2.5 × 10^5^, 1 × 10^5^, and 5 × 10^4^ cells per well, respectively) and were then cultured overnight. Cells were infected with STEC O113 WT or O113 Δ*subAB* at a multiplicity of infection (MOI) of 20 or with SubAB-expressing *C. rodentium* at an MOI of 10 in the presence or absence of SubABwt, SubABmt, or additives at the indicated concentrations. Plates were centrifuged for 10minat 700 ×*g* to synchronize the infection and were then incubated for 20 min. Cells were washed, and fresh medium containing 100 μg/mL of gentamicin was added to kill extracellular bacteria. After 2 h, the medium was changed to include 20 μg/mL of gentamicin with or without SubABwt, SubABmt, or additives, and the plates were incubated for an additional 16 h. To detect caspase-1 or caspase-11, culture supernatants were concentrated by means of methanol/chloroform precipitation, followed by dissolving with sodium dodecyl sulfate (SDS) sample buffer; cells were lysed with SDS sample buffer. For the bacterial survival assay, the infected monolayers were lysed in the tissue culture dishes by means of the addition of 0.1% sodium deoxycholate in PBS. To determine bacterial counts, serial dilutions of lysates were plated onto BHI agar (Becton, Dickinson and Company) and were incubated overnight at 37°C. The number of viable bacteria was determined by using the CFU method, with the results shown as CFU per well.

#### LPS transfection (LPS TF)

J774.1 cells seeded in 24-well plates (1 or 2.5 × 10^5^ cells per well) were cultured overnight or transfected with siRNA for 72 h. Cells were stimulated for 4 h with 100 ng/mL LPS (LPS priming, 1^st^ LPS). SubABwt or SubABmt was added at 3 h after LPS priming. LPS/Lipofectamine2000 complexes were prepared as reported previously (Ruhl and Broz, 2015). For each well of primed cells to be transfected, 8 μg of LPS and 4 μL of Lipofectamine 2000 (Thermo Fisher Scientific) were mixed in 100 μL of Opti-MEM (Thermo Fisher Scientific). The transfection mixture was vortexed briefly, incubated for 15minat room temperature, and then added dropwise to the cells. After incubation for 14 h, culture supernatants were collected and cells were lysed with SDS sample buffer.

#### WB analysis

Culture supernatants or cell lysates in SDS sample buffer were heated at 98°C for 5 min, separated by means of SDS-PAGE, and transferred to polyvinylidene difluoride (PVDF) membranes (Merck Millipore, Darmstadt, Germany) at 100 V for 1 h, which were blocked with 5% of non-fat milk in TBS-T (20 mM Tris pH 7.6, 137 mM NaCl, and 0.1% Tween 20) for 1 h. The membranes were incubated with the indicated antibodies for 1hat room temperature or overnight at 4°C. After membranes were washed with TBS-T, they were incubated for 1 h with HRP-labeled secondary antibodies. After the membranes were washed again with TBS-T, bands were detected by using the Immobilon Western Chemiluminescent HRP Substrate (Merck Millipore) with the luminescent image analyzer ChemiDoc™ XRS system (Bio-Rad).

#### Immunoprecipitation (IP)

For the study of the formation of the inflammasome assembly, cells were plated in 24-well plates at a density of 2.5 × 10^5^ cells per well. After infection or treatment as indicated in the Figure Legends, cells were washed with ice-cold PBS, lysed with IP buffer (50 mM HEPES [pH 7.4], 1% Triton X-100, 10% glycerol, 150 mM NaCl, 1.5 mM MgCl_2_, 1 mM EGTA) containing 50 mM NaF, 1 mM Na_3_VO_4_, and protease inhibitor cocktail (cOmplete, Mini, EDTA-free) (Roche Diagnostics GmbH, Mannheim Germany), and then incubated on ice for 10 min. The samples were sonicated by using the Bioruptor UCD-250 (Tosho Electric, Tokyo, Japan) for 1 min at 10-s intervals and then centrifuged at 18,000 ×*g* for 10 min, followed by collection of supernatants as cytoplasmic protein extracts. Cytoplasmic protein extracts were incubated with protein G-Agarose (Thermo Fisher Scientific) to remove non-specific binding proteins. After incubation for 60 min on ice, supernatants were collected by centrifugation at 3,000 ×*g* for 2 min and were then incubated with anti-caspase-1 antibody at 4°C overnight. Immunocomplexes were collected by incubation with protein G-agarose at 4°C for 2 h, followed by centrifugation at 3,000 ×*g* for 2 min at 4°C. Immunocomplexes were washed with IP buffer three times, and proteins were dissolved and boiled for 5 min at 98°C in SDS sample buffer, subjected to SDS-PAGE, and then analyzed by using WB with anti-NLRP3, anti-caspase-1, and anti-caspase-11 antibodies.

#### ELISA

Mouse IL-1β, IL-18, or IFN-β in culture supernatants, or IL-18 in mouse tissue homogenates, was measured by using the mouse IL-1β/IL-1F2 Quantikine ELISA kit (R&D Systems), VeriKine Mouse IFN Beta ELISA kit (PBL Assay Science, Piscataway, NJ, USA), and Mouse IL-18 ELISA kit (MBL, Nagoya, Japan) according to the manufacturers’ instructions. Absorbance at 490 nm was then measured with an iMark Microplate Reader (Bio-Rad).

#### Quantitative real-time reverse transcription PCR (qRT-PCR) analysis

Total RNA was extracted from J774.1 cells by using the RNeasy Mini Kit (Qiagen), according to the manufacturer’s protocol. cDNA was synthesized by using a PrimeScript RT Master Mix (Takara Bio Inc., Shiga, Japan). qRT-PCR was performed on a ViiA7 system (Thermo Fisher Scientific) with TB Green Premix Ex Taq II (Takara Bio Inc.). Each melting curve was analyzed to confirm that the PCR signal was derived from a single PCR product. The amplification conditions consisted of an initial denaturation at 95°C for 20 s, followed by 40 cycles of denaturation at 95°C for 1 s and annealing/extension at 60°C for 20 s. A minimum of three separate samples was used, and the expression levels were calculated from at least two technical replicates. IL-1β mRNA expression levels were estimated by using the 2ΔΔCt method, and the mRNA levels were normalized to those of GAPDH mRNA. The primer sequences are listed in the key resources table (Fujiwara et al., 2018).

#### Reverse-transcription PCR (RT-PCR)

Total RNA was measured, and 1 μg was reverse transcribed by using the ReverTra Ace qPCR kit (TOYOBO, Osaka, Japan) according to the manufacturer’s instructions. cDNA was amplified by PCR by means of KOD FX polymerase (TOYOBO). The PCR conditions were as follows: initial denaturation at 94°C for 2 min, at 98°C for 10 s, at 50°C (IFN-β) or 55°C (GAPDH) for 30 s, and at 68°C for 30 s, followed by a final step at 68°C for 5 min. Primers for PCR are given in the key resources table (Furukawa et al., 2011; Zhang et al., 2019). PCR products of 30 cycles (for GAPDH) or 35 cycles (for IFN-β) were subjected to electrophoresis on 2% agarose gels, and the bands were stained with ethidium bromide and visualized under UV light.

#### Lactate dehydrogenase (LDH) release assay

J774.1 cells seeded in 96-well plates were infected with STEC O113 WT or O113 Δ*subAB* at an MOI of 20 in the presence or absence of SubABwt or SubABmt. Culture supernatants were collected, and released LDH was measured by using a Cytotoxicity LDH Assay Kit-WST (Dojindo, Kumamoto, Japan) according to the manufacturers' instructions. Absorbance at 490 nm was then measured with an iMark Microplate Reader (Bio-Rad).

#### Mouse infection

Female 8-week-old C57BL/6J mice were purchased from Japan SLC Inc. (Shizuoka, Japan) and housed in the Center for Animal Resources and Development, Kumamoto University. Food and water intake was stopped 8 h before infection and was allowed to resume 1 h after infection. Mice were gavaged with PBS (Mock infection) or 5 × 10^9^ CFU of SubAB-producing Cr strains (Cr-SubABwt or Cr-SubABmt) or the pET23b(+) empty vector control strain (Cr-Empty) in 100 μL of PBS. Body weights were monitored daily, and mice were euthanized if they lost >25% of their body weight as a humane endpoint and were scored as dead. Survival curves were constructed by using the Kaplan-Meier method, and statistical significance was analyzed via the log-rank (Mantel-Cox) test with GraphPad Prism 7.0 (GraphPad Software, La Jolla, CA, USA). To analyze the number of bacteria in feces, mice from each infection group were euthanized at 6 and 11 days post infection (dpi); feces were randomly collected from intestines and fecal weights were measured. To determine bacterial counts, serial dilutions of fecal slurries (20% w/v in PBS) were plated onto MacConkey agar (Nissui Pharmaceutical Co., Ltd, Tokyo, Japan) containing ampicillin (100 μg/mL) and were incubated overnight at 37°C. The number of viable bacteria was determined by the CFU method, with the results shown as CFU per gram of feces.

#### Tissue collection and histology

Necropsy was performed at 14 and 15 dpi for Cr-SubABwt-infected mice and at 16 dpi for Mock and Cr-SubABmt-infected mice. Liver samples were fixed in 10% neutral buffered formalin and were embedded in paraffin. After the tissues were sectioned (3 μm thick), paraffin-embedded tissues were used for the immunostaining of macrophages and neutrophils with rabbit polyclonal anti-Iba1 antibody (Fujifilm Wako Pure Chemical Corp.) and rat monoclonal anti-Gr-1 antibody (SouthernBiotech), respectively. Sections were subsequently treated with Histofine Simple Stain MAX-PO (Rabbit) or Histofine Simple Stain Mouse MAX PO (Rat) (Nichirei, Tokyo, Japan). Reactions were visualized by using diaminobenzidine (DAB) solution (425011, Histofine DAB Substrate Kit; Nichirei Bioscience). Iba1- and Gr-1-positive cells were counted in randomly selected areas of high-power fields of a microscope by two observers blinded to the conditions.

#### Tissue protein isolation and analysis of BiP cleavage, protein expression, and IL-18 production

Intestinal tissues from infected mice were homogenized and protein was extracted by using RIPA buffer (10 mM Tris-HCl [pH 7.5], 1% NP-40, 0.1% SDS, 0.1% sodium deoxycholate, 150 mM NaCl, 50 mM NaF, 1 mM Na_3_VO_4_, and protease inhibitor cocktail). Briefly, a 5× volume of RIPA buffer was added to a sample, which was homogenized by means of a Polytron homogenizer on ice and was then centrifuged at 13,000 ×*g* for 10 min at 4°C. The supernatant was collected and the protein concentration was determined by using the Protein Assay BCA Kit (Fujifilm Wako Pure Chemical Corp.). For BiP cleavage and protein expression assays, 20-μg samples were subjected to SDS-PAGE and then analyzed by using WB with anti-BiP, anti-caspase-1, anti-caspase-11, and anti-IL-1β antibodies. For IL-18 analysis, protein concentration was adjusted to 50 μg/mL with assay diluent of the mouse IL-18 ELISA kit (MBL). IL-18 levels were determined by using the Mouse IL-18 ELISA kit according to the manufacturer’s instructions. Absorbance at 490 nm was then measured with an iMark Microplate Reader (Bio-Rad).

### Quantification and statistical analysis

Student's *t*-test was used to determine significant differences when only two treatment groups were being compared. All data are given as means ± standard deviation (SD).Data for each experiment were acquired from at least three experiments. A *p*-value of less than 0.05 was considered to be statistically significant.

## Data Availability

This study did not generate any unique datasets or code.
