# Supplementary material for: Subtilase cytotoxin from Shiga-toxigenic Escherichia coli impairs the inflammasome and exacerbates enteropathogenic bacterial infection
Source: iScience. 2022 Mar 10;25(4):104050. doi: 10.1016/j.isci.2022.104050 (PMC8957020; doi:10.1016/j.isci.2022.104050)
Supplement: Document S1. Figures S1–S6 [file mmc1.pdf]

iScience, Volume *25*

Supplemental information

Subtilase cytotoxin from Shiga-toxigenic *Escherichia coli* impairs the inflammasome and exacerbates enteropathogenic bacterial infection

Hiroyasu Tsutsuki, Tianli Zhang, Kinnosuke Yahiro, Katsuhiko Ono, Yukio Fujiwara, Sunao Iyoda, Fan-Yan Wei, Kazuaki Monde, Kazuko Seto, Makoto Ohnishi, Hiroyuki Oshiumi, Takaaki Akaike, and Tomohiro Sawa

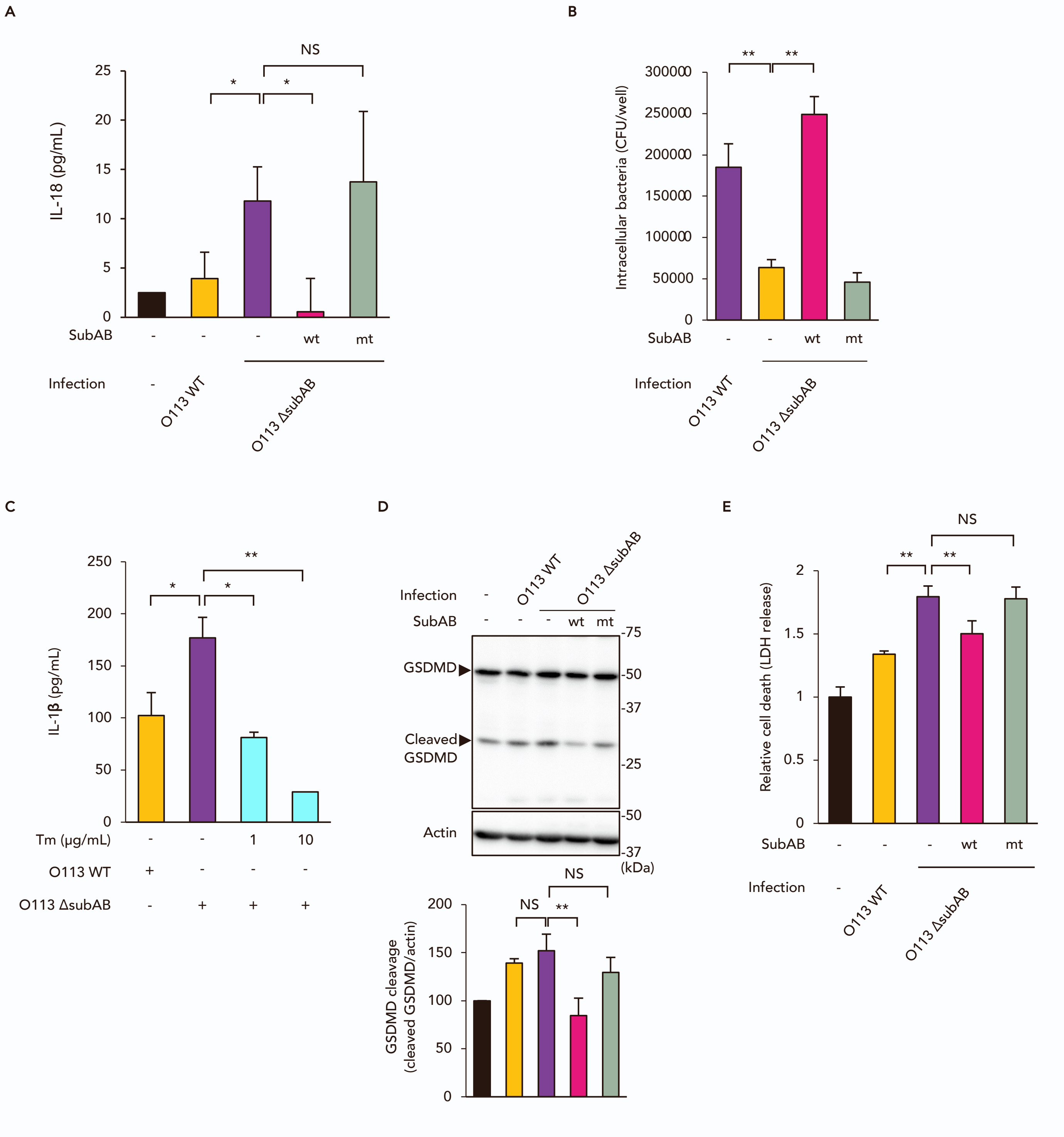

Figure S1. SubAB inhibits production of IL-1β and IL-18 and induction of pyroptotic cell death, related to Figure 1.
(A, B, D, and E) J774.1 cells were infected (MOI = 20) with SubAB-positive STEC O113 (O113 WT) and SubAB-deficient STEC O113 (O113 ΔsubAB) bacteria in the presence or absence of SubABwt or SubABmt for 16 h. (A) Culture supernatants were analyzed for IL-18 by using ELISA. (B) Intracellular bacteria were quantified by using CFU. (C) Culture supernatants of J774.1 cells that had been infected as indicated for 16 h in the presence or absence of tunicamycin (Tm) were analyzed for IL-1**β** by using ELISA. (D) Cell lysates were analyzed by Western blotting (WB) using anti-GSDMD and anti-actin antibodies. (E) Culture supernatants were analyzed for LDH release. Data are means ± SD (n=3-5). **p* < 0.05; ***p* < 0.01; NS, not significant.

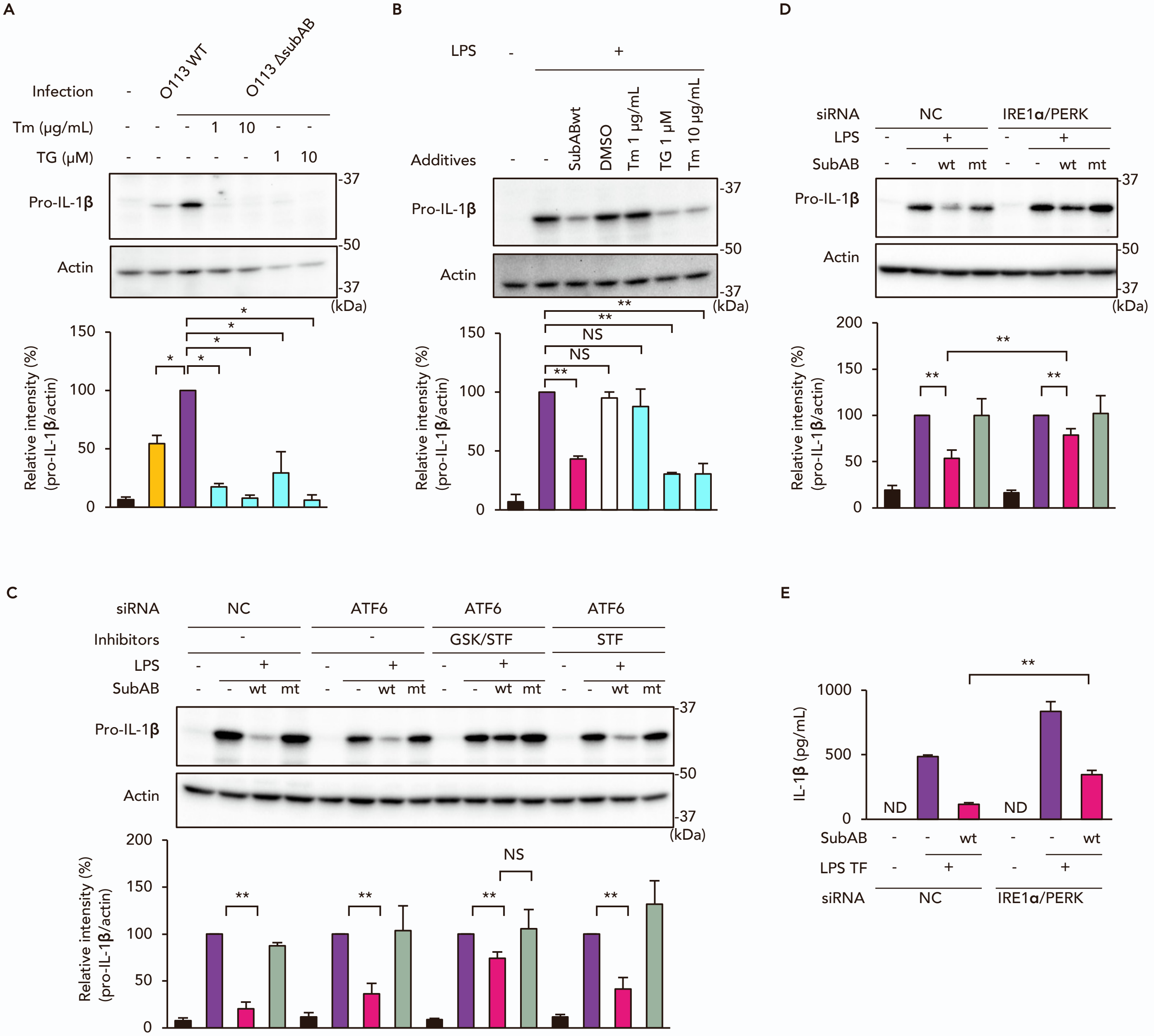

Figure S2. SubAB inhibits pro-IL-1β expression via PERK- and IRE1α-dependent pathways, related to Figure 2.
(A) J774.1 cells that had been pre-treated with the indicated concentrations of TG or Tm for 1 h were infected (MOI = 20) with O113 WT or O113 ΔsubAB for 16 h. Cells were analyzed by using WB with anti-IL-1β antibody. (B) Cells were pre-treated with Tm (1, 10 μg/mL) or TG (1 μM) for 1 h or were not so treated. Cells were then treated with LPS (100 ng/mL) in the presence or absence of SubABwt for 4 h. (C) Cells were transfected with negative control (NC) siRNA or ATF6 siRNA for 72 h. Cells were pre-treated with 1 μM GSK or 100 μM STF and were then treated with LPS for 4 h with or without SubABwt or SubABmt. Total cell lysate samples were analyzed by using WB with anti-IL-1β and anti-actin antibodies. Band intensity in each case was analyzed by means of densitometry, and results appear under the WB images. (D) NC or IRE1α/PERK knockdown cells were treated with LPS for 4 h with or without SubABwt or SubABmt. (E) NC or IRE1α/PERK knockdown cells were treated with LPS TF with or without SubABwt. Culture supernatants were subjected to ELISA for IL-1β. Data are means ± SD (n = 3). *p < 0.05; **p < 0.01; NS, not significant; ND, not detected.

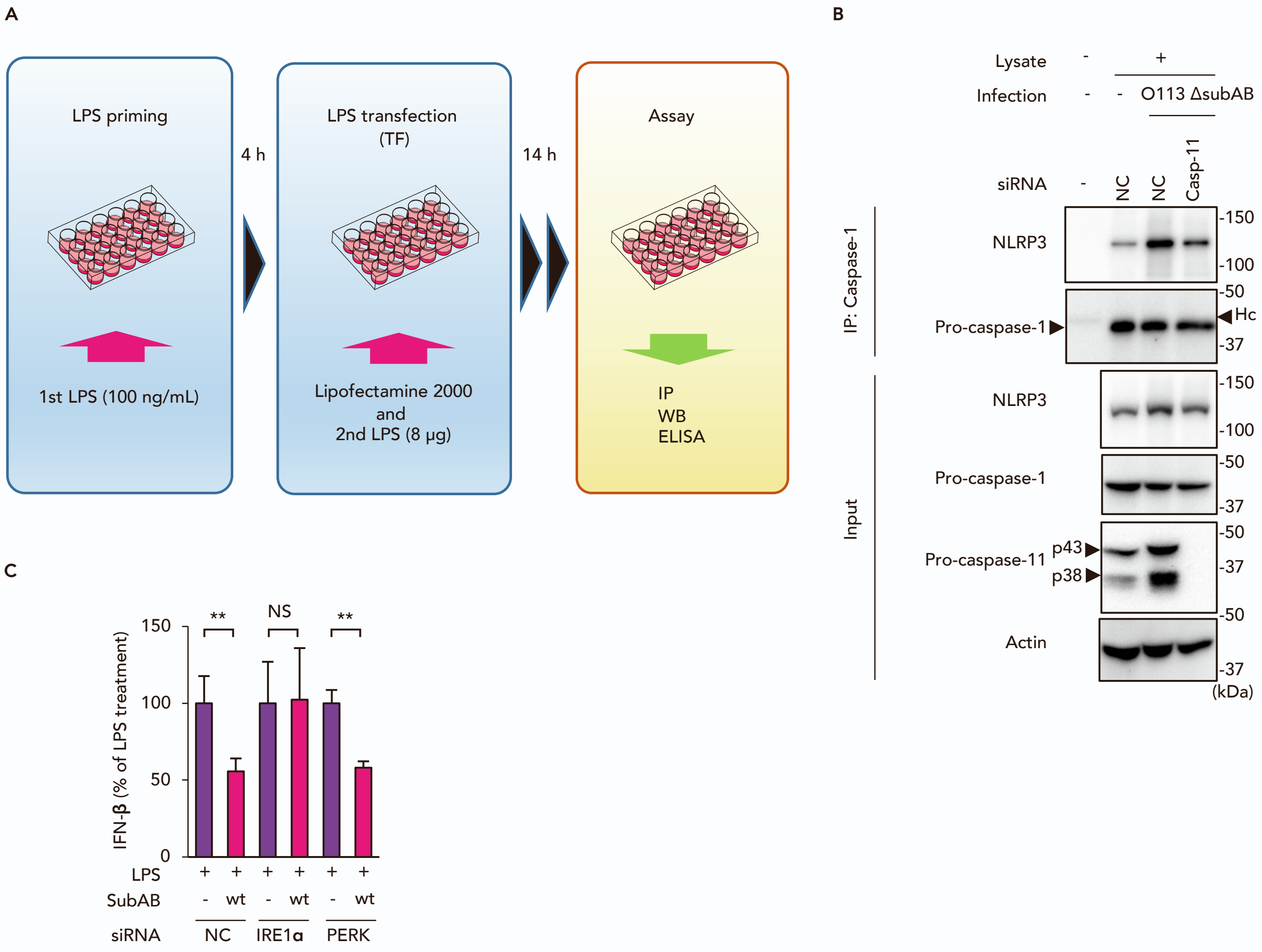

Figure S3. SubAB inhibits formation of the NLRP3 inflammasome complex and IFN-β production, related to Figure 2, Figure 3, and Figure 4.
(A) J774.1 cells in a 24-well plate were treated with 100 ng/mL LPS (LPS priming) for 4 h. Cells were transfected with 8 μg of LPS by means of Lipofectamine 2000. After 14 h, total cell lysate samples and supernatant were analyzed by using IP, WB, or ELISA. (B) J774.1 cells were transfected with NC siRNA or caspase-11 siRNA. Cells were infected with STEC O113 WT or O113 ΔsubAB (MOI = 20) for 16 h, and total cell lysate samples were analyzed by using IP with anti-caspase-1 antibody. NLRP3 binding was analyzed by using WB with anti-NLRP3 antibody. Hc indicates the heavy chain of anti-caspase-1 IgG. (C) NC, IRE1α or PERK knockdown cells were treated for 6 h with additives as indicated. IFN-β production was analyzed by ELISA. Data are means ± SD (n = 3). ** $p < 0.01$; NS, not significant.

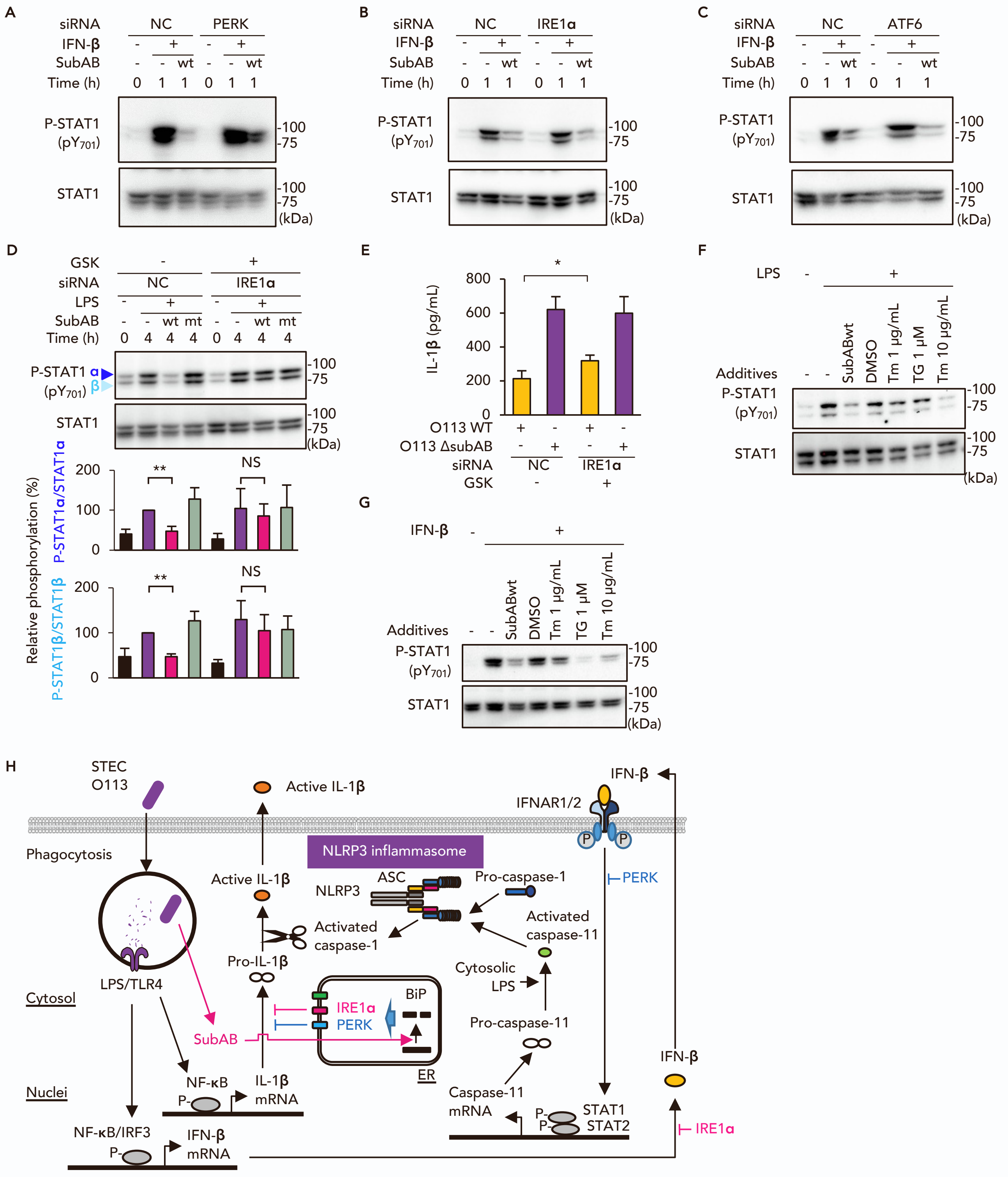

Figure S4. SubAB inhibits caspase-11 expression through PERK-mediated attenuation of IFN-β-induced STAT1 phosphorylation, related to Figure 5.

(A-C) J774.1 cells transfected with siRNA for 72 h were treated with or without SubABwt or SubABmt. After 3 h, cells were stimulated with or without IFN-β (100 pg/mL) for 1 h. (D) Cells transfected with NC siRNA or IRE1α siRNA for 72 h were pre-treated with or without 1 μM GSK for 1 h. Cells were then stimulated with or without LPS (100 ng/mL) for 4 h in the presence or absence of SubABwt or SubABmt. (E) Cells transfected with NC siRNA or IRE1α siRNA for 72 h were pre-treated with 1 μM GSK for 1 h or were not treated. Cells were then infected with STEC O113 WT or O113 ΔsubAB (MOI = 20) for 16 h, and culture supernatants were analyzed by ELISA for IL-1β. (F and G) Cells were treated with additives as indicated and stimulated with LPS for 4 h (F) or IFN-β for 1 h (G). Data are means ± SD (n = 3). *p < 0.05; **p < 0.01; NS, not significant. (H) Proposed model for SubAB-mediated inhibition of the non-canonical NLRP3 inflammasome and IL-1β generation through the ER stress sensors PERK and IRE1α in macrophages. IRF3, interferon regulatory transcription factor.

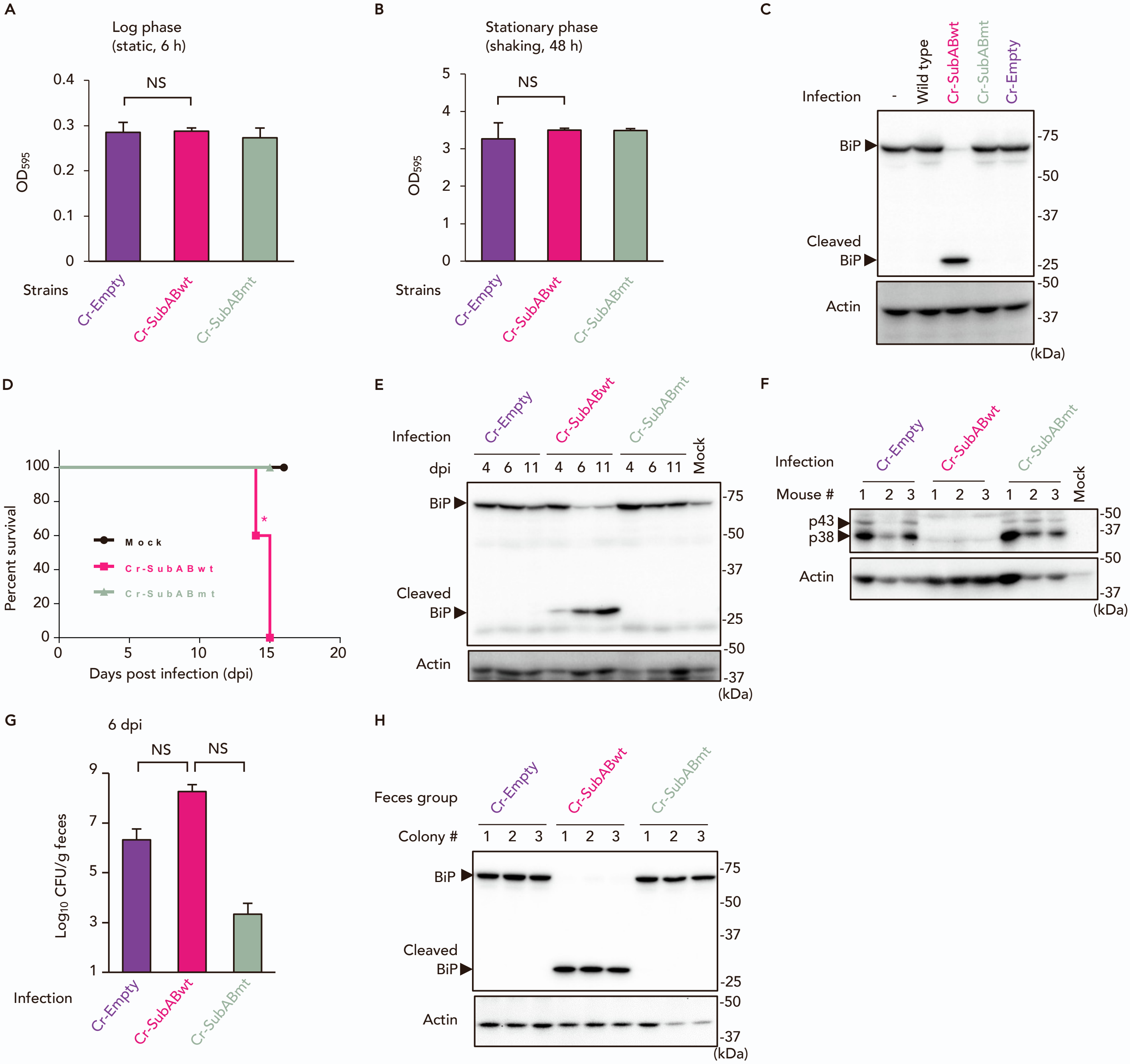

Figure S5. SubAB inhibits intestinal caspase-1 activation and production of IL-1β and IL-18 and promotes intestinal survival of *C. rodentium*, related to Figure 6.
(A and B) Bacterial growth of *C. rodentium* strains in LB medium. Bacterial growth was measured at 595 nm (OD_{595}) during the log phase (A) and the stationary phase (B). (C) J774.1 cells were infected (MOI = 20) with *C. rodentium* strains prepared in Fig 6A. Total cell lysate samples were analyzed by using WB with anti-BiP antibody. (D) Percent survival of groups of five mice that were infected as shown in Figure 6B. (E) BiP cleavage at 4-11 dpi in intestines of mice that were infected with vector control *C. rodentium* (Cr-Empty), Cr-SubABwt, or Cr-SubABmt. Tissue homogenates of intestines from infected mice were analyzed by using WB with anti-BiP antibody. (F) Intestinal homogenates from infected mice at 11 days post infection (dpi) were subjected to WB with anti-caspase-11 (p43 and p38) and anti-actin antibodies. (G) The number of viable bacteria in mouse intestinal contents was determined at 6 dpi by means of the CFU assay. Data are means ± SD (n = 3 per group). (H) WB for BiP cleavage in cultured J774.1 cells after macrophage infection of cells with Cr strains isolated from mouse feces (at 11 dpi). Bacterial colonies on MacConkey agar plates after the CFU assay as shown in Fig 6F were inoculated into LB broth-Amp. Cultures were calculated bacterial number (CFU/mL) and infected with J774.1 cells for 16 h (MOI = 20). BiP cleavage was analyzed by using WB with anti-BiP antibody. *$p < 0.05$; NS, not significant. Actin (C, E, F, and H) were served as the loading control for WB.

A

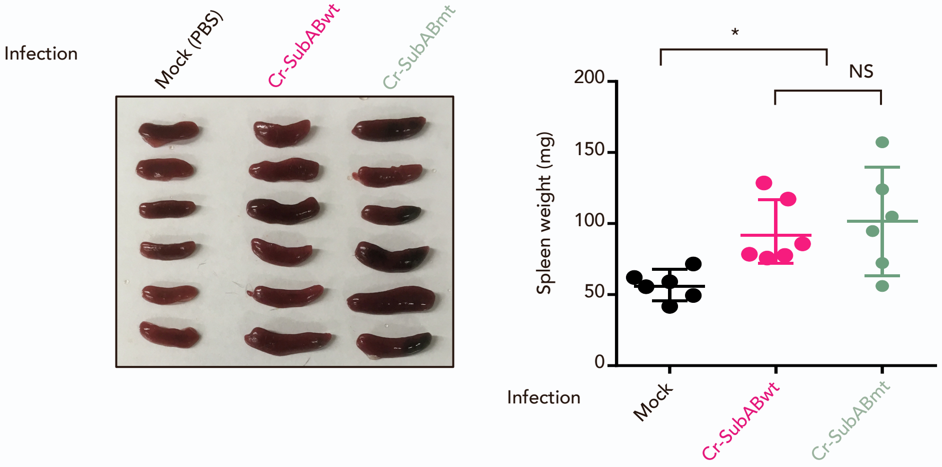

B

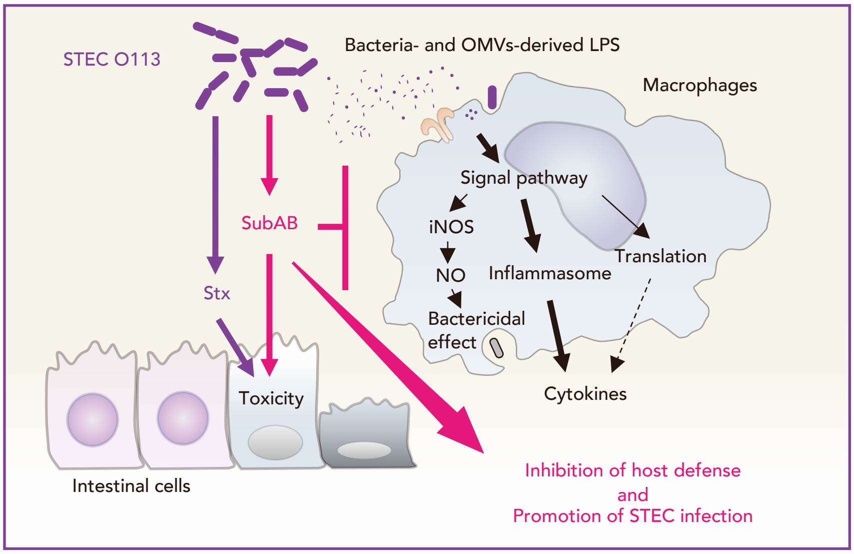

Figure S6. SubAB facilitates enteropathogenic bacterial infection in the mouse, related to Figure 6.
(A) Representative gross images of spleens from mice at 15-16 dpi that had been treated with PBS or infected with Cr-SubABwt or Cr-SubABmt. The right panel provides the quantitative weights of spleens that are shown on the left. Data are means ± SD (n = 6). *$p < 0.05$; NS, not significant. (B) A proposed model for promotion of the pathogenicity of SubAB-producing STEC by the impaired host defense system.
